# Leaf bidirectional reflectance distribution function (BRDF) prediction with phenotypic traits in four species: Development of a novel measuring and analyzing framework

**DOI:** 10.1016/j.plaphe.2025.100135

**Published:** 2025-10-30

**Authors:** Liangchao Deng, Leo Xinqi Yu, Linxiong Mao, Yanjie Wang, Xiyue Guo, Minjuan Wang, Yali Zhang, Qingfeng Song, Xin-Guang Zhu

**Affiliations:** a The Key Laboratory of Oasis Eco-agriculture, Xinjiang Production and Construction Group, Shihezi University, Shihezi, 832003, China; b Key Laboratory of Plant Carbon Capture, CAS Center for Excellence in Molecular Plant Sciences, Institute of Plant Physiology and Ecology, Chinese Academy of Sciences, Shanghai, 200032, China; c Key Lab of Smart Agriculture Systems, Ministry of Education, College of Information and Electrical Engineering, China Agricultural University, Beijing, 100083, China

**Keywords:** BRDF, Leaf phenotypic traits, Canopy light distribution, Ensemble learning model, Photosynthetic efficiency

## Abstract

Light intensity and spectral distribution within plant canopies provides insights into the effects of optimizing canopy architecture on light use efficiency. Breeding crop varieties with a “smart” canopy, characterized by erect upper-layer leaves and flat lower-layer leaves, can be supported with a 3D canopy model which can simulate light distribution for a particular canopy architecture. Leaf optical properties are required parameters for such canopy photosynthesis model to accurately predict canopy microclimate and hence photosynthetic efficiency. In this study, we developed a strategy to estimate the leaf optical properties based on leaf anatomical features. We developed a Directional Spectrum Detection Instrument (DSDI) system and associated Bidirectional Reflectance Distribution Function (BRDF) analysis software to precisely describe leaf light distribution. BRDF parameters were quantified with high accuracy ($R^{2}>\;0.95$) for adaxial and abaxial surfaces of maize, rice, cotton, and poplar leaves across canopy layers. Leaf phenotypic traits, surface roughness, pigments content, specific leaf weight and thickness were also assessed. Ensemble learning (EL) model showed excellent predictive performance for leaf optical properties based on phenotypic traits with R^2^ between 0.83 and 0.99. Compared to existing BRDF measurement systems, the DSDI achieves broader angular coverage (-π/36 to 35π/36) via mechanical rotation design, and the ensemble learning model establishes the first direct predictive relationship between BRDF parameters and leaf phenotypic traits. This work presents a new approach to quantify leaf optical properties and offers predictive models for leaf optical properties, which can support canopy light distribution prediction and hence support design leaf features for higher canopy photosynthesis efficiency.

## 1 Introduction

Canopy photosynthesis is the sum of the photosynthesis of all aboveground tissues, which correlates with biomass accumulation and improving canopy photosynthesis is a major breeding target for crop high light use efficiency [1]. Canopy architecture primarily determines the absorption and distribution of solar light within a canopy [2] and controls the use of light energy to achieve a greater canopy photosynthetic rate [3,4]. The intensity of light and spectral signals, such as red/far red ratio, regulates plant morphology, which also influences canopy photosynthesis [5]. Improving plant architecture for an optimal canopy architecture under higher planting density is widely used in crop breeding and cultivation for higher crop yield [6,7]. The rice ideotype which includes erect top leaves and medium number of tillers has been widely applied in breeding [8]. The maize “smart” canopy with small leaf angle for top leaves and large leaf angle for bottom leaves is suitable for higher planting density, which is a major contributor to maize yield in the past decades [9]. Similarly in soybean, plants with compact structures and narrower leaves are adapted for high density planting for higher yield [10,11]. Plant architectural traits, including plant height and leaf angles, are also extensively optimized for other crops, such as wheat and cotton [12,13]. Though with these successes, designing and optimizing canopy architecture still represents a major target for current crop breeding. Studying genetic mechanisms underlying various plant architectural features is also a major research area in current plant biology research community.

By constructing a 3D canopy photosynthesis model, optimal plant architecture can be studied [14,15]. 3D canopy models can be built based on either mathematical models parameterized with plant structural parameters [16], data directly obtained from 3D plant phenomics platform, such as multi-view stereo imaging [17,18] and lidar [19,20]. Accurately characterization of light distribution within a canopy is critical for designing ideal canopy architecture for higher canopy photosynthesis efficiency [21,22]. Ray tracing algorithm has been used to effectively simulate the absorbed light, transmitted light, and reflected light after light ray reaches the leaf surface [23,24]. The spatial distribution pattern of the transmitted and reflected light, which is determined by leaf optical properties, is essential for the accurate prediction of light environment inside a canopy with a ray tracing algorithm.

Leaves mainly absorb visible light between wavelengths 400–700 ​nm. The absorption coefficient is usually higher than 0.9 for blue photons and higher than 0.7 for green photon. The pigment content has a major influence on the absorption coefficient. Leaves absorb less infrared light with wavelength 700–1000 ​nm [25,26]. The spatial distribution patterns of reflected infrared light is more uniform than those for visible light [27]. Leaf surface roughness and pigment content significantly influence the distribution of reflected light [28,29]. Leaf surface roughness varies among plant species, e.g., the roughness of rice leaf is high while the roughness of cotton leaf is low. The light incident angle also affects the distribution of reflected light [30]; leaf angle similarly influences light distribution in canopy. The adaxial and abaxial surfaces have different optical properties due to the cellular anatomy of leaf section and the chlorophylls distributions inside a leaf, which differ dramatically between flat and vertical leaves [31].

Although recent advances in 3D canopy modeling and smart breeding have highlighted the critical role of leaf optical properties in regulating canopy photosynthesis, practical evaluation of these parameters remains challenging. Existing canopy photosynthesis models often incorporate radiative transfer but typically assume uniform optical properties among leaves [32], overlooking the variability caused by environmental conditions and developmental stages, which reduces the predictive power of canopy photosynthesis models in both mechanistic studies and breeding applications. Optical instruments can directly measure leaf reflectance and transmittance [33], yet these measurements are slow and impractical for large-scale phenotyping. This limitation hampers the integration of leaf-level optical diversity into large-scale phenotyping and canopy photosynthesis modeling. In contrast, predicting optical parameters from measurable biochemical and structural traits offers a rapid alternative for characterizing leaf optical diversity and improving the parameterization of canopy photosynthesis models. Because anatomical structures and pigment compositions fundamentally determine leaf scattering and absorption [34], establishing quantitative relationships between these traits and optical parameters provides a scalable and efficient means of estimating leaf optical behavior across species and canopy positions.

The spatial distributions of reflected light and transmitted light, can be described with a Bidirectional Reflectance Distribution Function (BRDF), which have specific parameters including roughness (σ(λ)), diffuse reflection coefficient (k(λ)) and refractive index (n(λ)) [35]. A variety of specialized equipment have been developed to sample the bidirectional and spectral reflectance of leaves and interpreted the spectral and directional variations in leaf reflectance with BRDF [36,37]. Among these parameters, σ(λ) is primarily affected by epidermal micromorphology and surface irregularities [38]; k(λ) depends on mesophyll scattering related to leaf thickness and internal spaces [39]; and n(λ) is influenced by biochemical composition [40]. Therefore, BRDF parameters are inherently linked to leaf anatomy, pigment composition, and physiological traits. Predicting BRDF parameters from leaf anatomy and physiological traits can be a potential efficient method. However, the quantitative relationships between BRDF parameters and these leaf traits remain poorly understood, and no predictive model currently exists for estimating BRDF parameters directly from leaf anatomical and physiological data.

To bridge this gap, we propose a scalable, phenomics-oriented approach to quantify leaf optical properties from measurable anatomical and biochemical traits. We developed a Directional Spectrum Detection Instrument (DSDI) that allows efficient measurement of leaf Bidirectional Reflectance Distribution Function (BRDF) across a broad range of illumination and viewing angles. The BRDF model was parameterized using roughness (σ(λ)), diffuse reflection coefficient (k(λ)), and refractive index (n(λ)), which link physical surface scattering and internal absorption processes to measurable leaf traits. Moreover, we established an ensemble learning (EL) framework to predict BRDF parameters based on leaf phenotypic traits such as thickness, specific leaf weight, pigment content, and surface roughness. This integration of optical measurement, modeling, and data-driven prediction establishes a new pathway toward computational phenotyping of optical traits, facilitating the parameterization of 3D canopy models for photosynthesis simulation.

## 2 Materials and methods

### 2.1 Experimental design

**Fig. 1:**
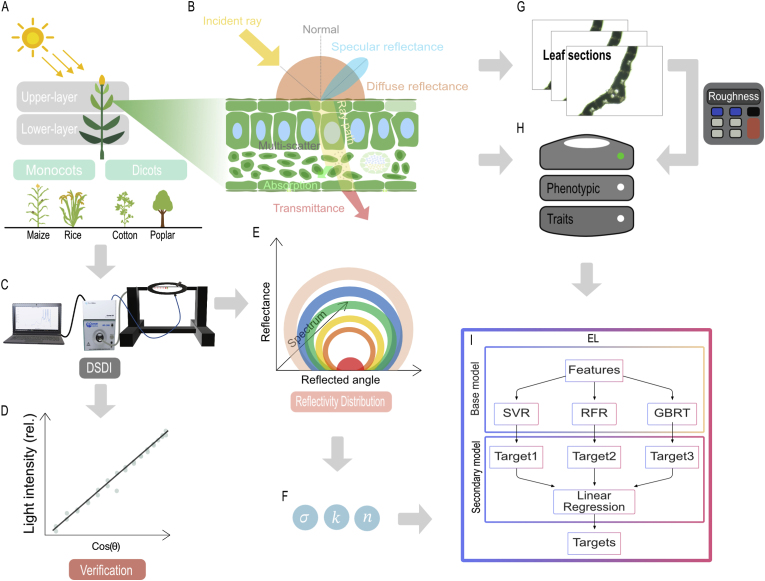
Schematic of the experimental design and the development of the predictive framework for optical properties. The upper- and lower-layer leaves from four plant species (maize, rice, cotton, and poplar), categorized into monocots and dicots, were used (A). Light was absorbed by a leaf and reflected and transmitted from the leaf. The reflect light includes specular and diffuse portion, and this reflect light distribution can be modeled with BRDF (B). Leaf section microscopy images were analyzed to obtain surface roughness data (G), which, along with other phenotypic traits (H), were fed into a predictive model. The DSDI platform was developed for measuring leaf reflect light distribution (C), calibrated for data accuracy with white board standard (D). Data of anatomical and physiological traits and the reflect light distribution data were used to develop ensemble learning (EL) model, including Support Vector Regression (SVR), Random Forest Regression (RFR), and Gradient Boosting Regression Tree (GBRT), for accurate prediction of BRDF parameters, roughness (σ(λ)), diffuse reflection coefficient (k(λ)) and refractive index (n(λ)). This study develops the BRDF parameter acquisition tools and its prediction model based on the data of leaf anatomical and physiological traits, which supports canopy light-use efficiency modeling.

This study investigated the relationships between leaf phenotypic traits and Bidirectional Reflectance Distribution Function (BRDF) parameters for leaves from different plant species and from different canopy layers with a goal of developing a predictive framework for leaf optical properties. As shown in Fig. 1, four plant species including maize (*Zea mays* L.), rice (*Oryza sativa* L.), cotton (*Gossypium hirsutum* L.) and poplar (*Populus alba* L.) were used in this study. The adaxial and abaxial surfaces of these leaves from both upper and lower layer of canopies were measured. Reflectance light distribution was measured with a custom-built Directional Spectrum Detection Instrument (DSDI), calibrated using a diffuse whiteboard standard (WS-1, PTFE-based Lambertian material; Ocean Insight Inc., USA) with reflectivity greater than 98 ​% across 250–1500 ​nm. Leaf optical property related traits, including chlorophyll *a* (Chl.a), chlorophyll *b* (Chl.b), carotenoid content (Car.), specific leaf weight (SLW), and thickness (T), were quantified with established protocols. Leaf surface roughness was determined using a custom developed image processing software, Roughness Calculator (RC), based on the leaf section microscopy images. Symbols used in the paper are shown in Table 1.

**Table 1:** Description of symbols used in the paper.

Symbol	Quantity	Unit(symbol)
L	Illumination direction vector	None
V	Viewing direction vector	None
N	Normal to the sample vector	None
H	Diagonal vector of L and V	None
λ	Wavelength	Nanometer (nm)
θ	Zenith angle	Degree (°)
φ	Azimuth angle	Degree (°)
α	Angle between N and H	Degree (°)
$\theta_{h}$	Half of the phase angle between L and V	Degree (°)
$f_{brdf}$	Bidirectional reflectance	Unit per steradian ($sr^{-1}$)
$f_{samp}$	Bidirectional reflectance of sample	Unit per steradian ($sr^{-1}$)
$f_{ref}$	Bidirectional reflectance of reference	Unit per steradian ($sr^{-1}$)
$f_{spec}$	Reflectance of specular component	Unit per steradian ($sr^{-1}$)
$f_{diff}$	Reflectance of diffuse component	Unit per steradian ($sr^{-1}$)
dA	Unit area	Square meter ($m^{2}$)
dΩ	Unit solid angle	Steradian (sr)
ϕ	Radiant flux	Watt (W)
$L_{r}$	Radiance	Watt per square meter per steradian ($W\cdot m^{-2}\cdot sr^{-1}$)
E	Irradiance	Watt per square meter ($W\cdot m^{-2}$)
σ(λ)	Roughness	None
k(λ)	Diffuse reflection coefficient	None
n(λ)	Refractive index	None
Chl.a	Content of leaf chlorophyll *a*	$\left(mg\cdot {dm}^{-2}\right)$
Chl.b	Content of leaf chlorophyll *b*	$\left(mg\cdot {dm}^{-2}\right)$
Chl.a+b	Total chlorophyll content (sum of Chl.a and Chl.a)	$\left(mg\cdot {dm}^{-2}\right)$
Car.	Leaf carotenoid content	$\left(mg\cdot {dm}^{-2}\right)$
T	Leaf thickness	(mm)
SLW	Specific leaf weight	($g\cdot m^{-2}$)
ρ	Leaf roughness measured by section	None

Subscripts λ, i and v represent the wavelength, illumination and viewing directions.

To analyze the effects of species and canopy layer on leaf phenotypic traits, two-way ANOVA was conducted using R software (version 4.5.1; R Core Team, 2023). The analysis was implemented with the *car* package for Type III sum-of-squares ANOVA [41], and post-hoc multiple comparisons were performed using the *emmeans* [42] and *rstatix* packages [43]. Independent *t*-test for group comparison was conducted in Excel (Microsoft Corporation, Redmond, WA, USA, version 365). BRDF parameter fitting was performed in MATLAB (MathWorks Inc., Natick, MA, USA, version 2024b), ensuring a high precision in parameter estimation. Furthermore, an ensemble learning model was developed in Python (version 3.8; Python Software Foundation) with a *scikit-learn* library [44]. Model performance was assessed through cross-validation and evaluated using metrics including coefficient of determination $\left(R^{2}\right)$ and mean square error (MSE) to validate predictive accuracy.

### 2.2 Plant materials

The experiment was conducted in 2021 ​at the Institute of Plant Physiology and Ecology, Chinese Academy of Sciences (CAS), Shanghai, China. Four plant species were used in this study including maize, rice, cotton and poplar. All plants were grown in a greenhouse with controlled environment, day/night temperatures of 25/18 ​°C and a relative humidity of 60–70 ​%. At the time of measurement, maize plants were approximately 2.0 ​m tall at the silking stage, rice plants at the heading stage were about 0.9 ​m tall, cotton plants at the boll-forming stage were about 1.5 ​m tall, and poplar plants were about 2.0 ​m tall at the vigorous growth stage. To capture the variability in optical properties across canopy layers, leaves were sampled from both upper and lower canopy positions, defined as the upper and lower halves of the plant height, respectively. For each species, at least 3 plants were used and at least 6 leaves from each plant were used for the measurements, with fully expanded leaves sampled from both upper and lower layers in canopy. For each leaf, both adaxial and abaxial surfaces were measured separately.

### 2.3 Ray tracing simulations for evaluation of canopy scatter light distribution

To quantify the effect of BRDF parameters on the spatial distribution of scattered light within plant canopies, ray tracing simulations were performed using a 3D rice canopy model (cultivar 9311 ​at the heading stage). The simulations were conducted with an optimized version based on the original ray tracing software (fastTracer, published by Song et al., 2013) [45]**.** The optimized version of fastTracer is available at https://github.com/PlantSystemsBiology/fastTracerPublic), which was further modified for this study to incorporate variable BRDF parameters. The 3D canopy model consisted of triangular leaf facets reconstructed from morphological measurements, representing the realistic spatial architecture of rice plants. Each photon was tracked through interactions with leaf surfaces, including reflection, transmit, and absorption, which were governed by the Cook–Torrance BRDF model.

To evaluate the individual and combined effects of the BRDF parameters, simulations were conducted under different combinations of leaf roughness (σ), diffuse reflection coefficient (k), and refractive index (n). The tested parameter sets included (σ, k, n) = (0.3, 0.01, 1.0), (0.3, 0.35, 1.0), (1.0, 0.01, 1.0), (0.3, 0.01, 2.2), (1, 0.35, 2.2) and (0.3, 0.35, 2.2). For each configuration, photons were emitted from the light source and traced until absorption or exit from the canopy domain. The resulting scattered photosynthetic photon flux density (PPFD) values were recorded at different canopy heights. The canopy space was divided into multiple horizontal layers of equal thickness, and the averaged PPFD were computed for each layer.

### 2.4 Development and evaluation of the DSDI system

**Fig. 2:**
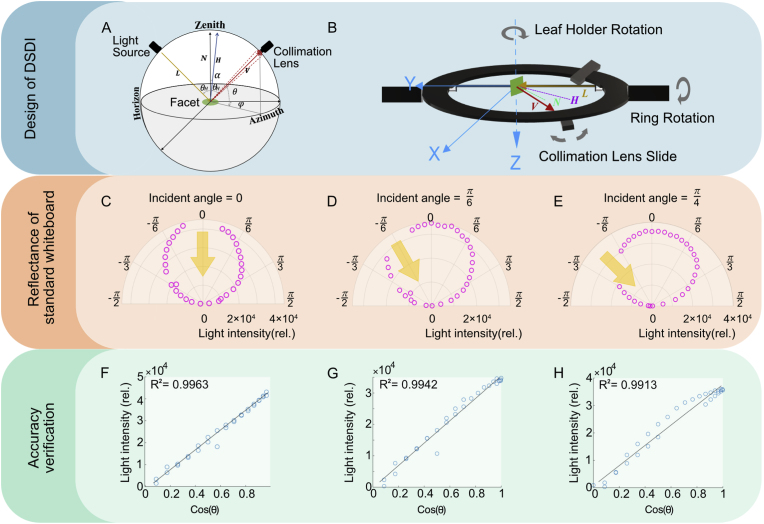
The design and verification of DSDI. A: the geometric design of the optical platform in DSDI, including rotating the leaf holder determining the illumination angle $\left(\theta_{i}{,\varphi }_{i}\right)$, rotating detection ring and sliding collimation lens determining the viewing or detecting angle $\left(\theta_{v},\varphi_{v}\right)$. B: the measurement diagram of DSDI. C–E: data of reflectance intensity measured with a standard whiteboard at incidence angles of 0, π/6 and π/4, and plotted in a polar coordinate system. Yellow arrows indicate the incident light direction. F–H: the linear relationship between reflectance intensity and cos(θ) at the three corresponding incidence angles (0, π/6 and π/4). θ represents the angle between the viewing direction and the normal.

The custom-built Directional Spectrum Detection Instrument (DSDI) is used to capture the angular spectrum from leaf surfaces (Fig. 1C). The DSDI setup incorporates an HPX-2000 high-power xenon light source (Detailed in Table S1) and an HR2000 high-resolution fiber optic spectrometer (Detailed in Table S2) (Ocean Insight Inc., USA). The distribution of reflectance on a leaf surface is typically characterized by the angular distribution of zenith (θ), and azimuth (φ) angles in spherical coordinates, visualized as the reflection hemisphere [46]. Fig. 2A illustrates the geometric relationship between incident and reflection angles, with the upper hemisphere representing the reflecting hemisphere. The measurement platform in DSDI has three axes (Fig. 2B), first, the leaf holder can be rotated with the *Z-*axis determining the illumination angle $\left(\theta_{i}{,\varphi }_{i}\right)$; second, the detection ring can be rotated with the *Y*-axis and third, the collimation lens can be slide on the detection ring. The detection ring and the collimation lens together determines the detecting angle $\left(\theta_{v},\varphi_{v}\right)$. When leaf sample was placed into a leaf holder, the collimation lens then rotated around the leaf holder on a circular track, capturing measurements from multiple angles. For detailed information, see supplementary material (Figs. S1–S3).

To validate the accuracy of DSDI in the measurement of reflectance from different angles, we conducted tests using a Lambertian whiteboard with its reflectance following the Lambert cosine law, i.e., the reflect light intensity is linearly correlated with the cosine of the detection angle [47,48]. Reflect light intensity at different detection angles was measured and recorded for the whiteboard at incidence angles of 0, π/6 and π/4, respectively. (Fig. 2C–E). A linear relationship was derived between reflectance intensity and cosine of the detection angle ($R^{2}>\;0.99$) (Fig. 2F–H), confirming that DSDI provides reliable spatial light distribution measurements.

### 2.5 Measurement of spatial distribution of reflection spectrum of leaves

The directional distribution of transmitted light through leaves is physically similar to that of diffuse reflectance [33], and it can be approximated by a Lambertian function [49]. Therefore, this study focuses on the reflection distribution without separately analyzing the transmission component. The DSDI system was used to measure the reflection distribution of leaf as the following steps. Firstly, a leaf sample with area of 1cm×2cm was attached on the leaf holder. The leaf surface should be flat, and the position of the primary vein was not used. Secondly, the sample holder was rotated to set the incident angle. The light source was turned on, and a light spot can be observed on the leaf. The illuminated area is round for incident angle 0 and elliptical for other incident angles. Thirdly, the detection ring was rotated to be horizontal for measuring the reflection and then the collimation lens was slide along the detection ring to measure the reflect light spectrum at different reflection angles. Finally, the reflected light spectrum was measured and recorded by the spectrometer and the PC.

We collected reflectance data across a broad spectrum (400–992 ​nm) and selected five representative wavelengths (468.36, 556.26, 673.46, 819.88, and 877.97 ​nm) for further analysis as shown in Table 2. These wavelengths include the primary absorption (468.36 and 673.46 ​nm) for pigments such as Chl.a, Chl.b, and Car; the maximum reflectance peak in the green light region (556.26 ​nm); the near-infrared (NIR) region (819.88 and 877.97 ​nm).

**Table 2:** The selected wavelengths in the VIS-NIR spectrum.

Waveband	Blue	Green	Red	Near-infrared
Range (nm)	440–485	500–565	625–740	800–1300
Wavelength (nm)	468.36	556.26	673.46	819.88, 877.97

### 2.6 The definition of BRDF and its calculation based on the measured data with DSDI system

The Bidirectional Reflectance Distribution Function (BRDF) is used to describe the spatial reflecting characteristics of light on rough surfaces, such as the leaf surface [50]. The general bidirectional reflectance ($f_{brdf}$) can be defined as the ratio of radiance to irradiance, quantifying the contribution of the incident spectral irradiance from the direction $\left(\theta_{i},\varphi_{i}\right)$ to the reflected spectral radiance in the direction $\left(\theta_{v},\varphi_{v}\right)$ (Eq. (1)). Radiance is the radiant flux (power) per unit projected area per unit solid angle (unit: W·sr^−1^·m^−2^) and irradiance is the radiant flux incident on a surface per unit surface area.

(1) $$\begin{array}{c} f_{brdf}=\frac{L_{r}\left(\lambda ,\theta_{i},\varphi_{i},\theta_{v},\varphi_{v},\right)}{E\left(\lambda ,\theta_{i},\varphi_{i}\right)} \end{array}$$

The $f_{brdf}$ represents the bidirectional reflectance, *L* denotes radiance, *E* refers to irradiance, λ is the wavelength, $\theta_{i}$ is incident zenith angle, and $\varphi_{i}$ is the incident azimuth angle, $\theta_{v}$ is reflex zenith angle, and $\varphi_{v}$ is the reflex azimuth angle. The symbols and units used in this formula are summarized in Table 1.

According to the definitions, the radiance ($L_{r}$) can be derived with the measured reflected radiant flux ($\phi_{v}$) at a view angle ($\theta_{v}$) for a certain surface area (dA) and a certain solid angle (dΩ) as Eq. (2).

(2) $$\begin{array}{c} L_{r}=\frac{\phi_{v}}{dAcos\left(\theta_{v}\right)d\Omega } \end{array}$$

The irradiance (***E***) can be derived with the incident radiant flux ($\phi_{i}$) on the surface area (dA) with Eq. (3).

(3) $$\begin{array}{c} E=\frac{\phi_{i}}{dA} \end{array}$$

Substituting Eq. (2) and Eq. (3) into Eq. (1), we obtain Eq. (4) that describes the bidirectional reflectance for light at wavelength (λ) with incident direction $\left(\theta_{i},\varphi_{i}\right)$ and reflect direction $\left(\theta_{v},\varphi_{v}\right)$:

(4) $$\begin{array}{c} f_{brdf}\left(\lambda ,\theta_{i},\varphi_{i},\theta_{v},\varphi_{v}\right)=\frac{L\left(\lambda ,\theta_{i},\varphi_{i},\theta_{v},\varphi_{v}\right)}{E\left(\lambda ,\theta_{i},\varphi_{i}\right)}=\frac{\phi_{v}}{\phi_{i}\;\cos\left(\theta_{v}\right)d\Omega } \end{array}$$

Assuming that the reference whiteboard behaves as an ideal Lambertian surface with a hemispherical reflectance of 100 ​%, the bidirectional reflectance of the reference surface is 1/π [51]:

(5) $$\begin{array}{c} f_{ref}=\frac{\phi_{v,ref}}{\phi_{i}\;\cos\left(\theta_{v}\right)d\Omega }=\frac{1}{\pi } \end{array}$$

Thus, the bidirectional spectral reflectance of the leaf sample can be calculated relative to the whiteboard as following equations:

(6) $$\begin{array}{c} f_{samp}=\frac{\phi_{v,samp}}{\phi_{i}\;\cos\left(\theta_{v}\right)d\Omega }=\frac{\phi_{v,samp}}{\pi \cdot \phi_{v,ref}} \end{array}$$

Using the DSDI system, the measurement was performed for leaves and the reference whiteboard at several incident angles. For each incident angle (i), the $\phi_{v,samp}$ and $\phi_{v,ref}$ at various reflection angles were measured. Then the bidirectional reflectance for leaf sample ($f_{samp}$) could be calculated with Eq. (6).

In the practical operation of the DSDI system, all angular parameters are derived from the instrument's mechanical scales. Due to minor manufacturing and assembly deviations, the initial position of the leaf holder does not correspond to a true 0° orientation but instead to 95°, which represents the perpendicular illumination reference of the DSDI. Therefore, the leaf inclination angle $\theta_{leaf}$ was calculated with the scale of leaf holder ($\theta_{leafholder}$) as:

(7) $$\begin{array}{c} \theta_{leaf}=95{}^{\circ}-\theta_{leafholder} \end{array}$$

where 95° corresponds to the perpendicular illumination reference of the DSDI system.

To facilitate the calculation of reflection geometry, a three-dimensional Cartesian coordinate system was established (Fig. 2B). The illumination direction was defined as the positive direction of the *Y*-axis, while the *Z*-axis points vertically downward. The *X*-axis was defined according to the right-handed Cartesian coordinate system, perpendicular to the *YZ*-plane. Since the light source remained fixed, the illumination direction vector (L) was defined as:

(8) $$\begin{array}{c} L=\left(0,-1,0\right) \end{array}$$

Based on the leaf inclination angle, the leaf normal vector (N) can be expressed as:

(9) $$\begin{array}{c} N=\left(\sin\left(\theta_{leaf}\right),-\cos\left(\theta_{leaf}\right),0\right) \end{array}$$

During reflection measurements, the collimation lens slides along the detection ring to acquire reflectance at different angles. The ring's scale reading corresponds to the viewing zenith angle ($\theta_{v}$), while the rotation of the ring defines the viewing azimuth angle ($\varphi_{v}$). Accordingly, the viewing direction vector (V) can be calculated as:

(10) $$\begin{array}{c} V=\left(\sin\left(\theta_{v}\right)\cos\left(\varphi_{v}\right),-\sin\left(\theta_{v}\right),\sin\left(\theta_{v}\right)\sin\left(\varphi_{v}\right)\right) \end{array}$$

Based on the illumination direction vector (L) and the viewing direction vector (V), the half-vector (H) representing the bisector of illumination and viewing directions is calculated as:

(11) $$\begin{array}{c} H=\frac{L+V}{\sqrt{\left(L+V,L+V\right)}} \end{array}$$

Thus, all directional vectors and angular parameters required for the BRDF model, including the illumination, viewing, normal and half vectors, were determined geometrically within this coordinate framework.

### 2.7 The Cook-Torrance model for BRDF

In this study, we employ the classic Cook-Torrance model to calculate BRDF. The model was developed by Robert Cook and Kenneth Torrance in 1981 for surface with varying roughness based on geometric optics [52]. The reflectance of a rough surface lies between perfect diffuse and perfect specular reflectance, and can be expressed as the sum of the diffuse and specular components:

(12) $$\begin{array}{c} f_{brdf}=f_{spec}+f_{diff} \end{array}$$

For an ideal Lambertian surface, the bidirectional reflectance is 1/π [51]. The diffuse component of the bidirectional reflectance for a leaf surface can be expressed as Eq. (12) and the k(λ) is the diffuse reflection coefficient.

(13) $$\begin{array}{c} f_{diff}=\frac{k\left(\lambda \right)}{\pi } \end{array}$$

The specular reflection component is more complex than the diffuse reflection. The leaf surface can be approximated as a collection of micro-facets with irregular orientations [53], as shown in Fig. 1B. Specular reflection in this model is described as light reflecting from micro-facets, with the reflection occurring between the illumination and the viewing.

The leaf cuticle, which covers the epidermal cells, is considered a low-absorption medium compared to the leaf itself [54], allowing us to neglect its absorption. The light reflected from a single micro-facet can be defined by the Fresnel factor F(n,θ), which describes the proportion of non-polarized incident light reflected as specular reflection [55]:

(14) $$\begin{array}{c} F\left(n,\theta_{h}\right)=\frac{1}{2}{\left(\frac{n^{2}\left(\lambda \right)+\cos^{2}\left(\theta_{h}\right)-1-\cos\left(\theta_{h}\right)}{n^{2}\left(\lambda \right)+\cos^{2}\left(\theta_{h}\right)-1+\cos\left(\theta_{h}\right)}\right)}^{2}\left[1+{\left(\frac{\cos\left(\theta_{h}\right)\left(n^{2}\left(\lambda \right)+\cos^{2}\left(\theta_{h}\right)-1+\cos\left(\theta_{h}\right)\right)-1}{\cos\left(\theta_{h}\right)\left(n^{2}\left(\lambda \right)+\cos^{2}\left(\theta_{h}\right)-1-\cos\left(\theta_{h}\right)\right)+1}\right)}^{2}\right] \end{array}$$

The distribution of the micro-facet slopes is defined by the Beckmann distribution D(α,σ), which can be expressed as [56]:

(15) $$\begin{array}{c} D\left(\alpha ,\sigma \right)=\frac{e^{-{\left(\frac{\tan\left(\alpha \right)}{\sigma }\right)}^{2}}}{\sigma^{2}\;\cos^{4}\left(\alpha \right)} \end{array}$$

During illumination, micro-facets may shield and mask each other, causing light attenuation [37]. The geometric attenuation factor G(L,N,V,H) describes this effect, capturing the reduction of light due to multiple reflections between micro-facets [57]:

(16) $$\begin{array}{c} G\left(L,N,V,H\right)=\min\left(1,\frac{2\left(V,N\right)\left(N,H\right)}{\left(V,H\right)},\frac{2\left(L,N\right)\left(N,H\right)}{\left(V,H\right)}\right) \end{array}$$

Here, H is the angular bisector vector and can be calculated with Eq. (11) by assuming that H is the normal vector of the micro-facet [36].

In summary, the Cook-Torrance BRDF on the leaf surface can be expressed as:

(17) $$\begin{array}{c} f_{brdf}=\frac{F\left(n\left(\lambda \right),\theta_{h}\right)\cdot D\left(\alpha ,\sigma \left(\lambda \right)\;\right)\cdot G\left(L,N,V,H\right)}{2\pi^{2}\left(L,N\right)\left(N,V\right)}+\frac{k\left(\lambda \right)}{\pi } \end{array}$$

### 2.8 BRDF parameters fitting algorithms

According to the Cook-Torrance model of BRDF, three critical parameters (roughness (σ(λ)), diffuse reflection coefficient (k(λ)), and refractive index (n(λ))) of the BRDF determines leaf optical properties. These parameters influence reflectance distribution, which is vital for understanding light behavior on a leaf surfaces. The optimization of BRDF parameters was constrained by setting fixed upper and lower bounds for each parameter during the fitting process [36]. These bounds ensured that the parameter values remained within physically meaningful and biologically relevant ranges, thereby improving the robustness and accuracy of the optimization. The roughness, σ(λ), describes the surface texture and corresponds to the root mean square (RMS) of the slope of the micro-facets on the surface [49]. It ranges from 0 (perfectly smooth) to 1 (highly rough). A higher σ(λ) value indicates more irregular and scattered reflected light, while a lower value results in more directional and concentrated reflections [58]. The diffuse reflection coefficient, k(λ), represents the proportion of diffuse light reflected from the surface, with values between 0 and 1. The k(λ) value of 0 indicates no diffuse reflection, while a value of 1 suggests complete diffuse reflectance. The refractive index, n(λ), quantifies the extent to which light attenuates when passing through the leaf medium and typically ranges from 1 to 5 for leaves [59].

To accurately characterize the BRDF parameters of leaf surfaces, we employed two fitting methods: Least Squares curve fitting and Adaptive Grid Search. The least squares method is a traditional regression technique that estimates parameter values by minimizing the sum of squared residuals between observed and fitted values. Using MATLAB's least squares fitting function, we estimated the BRDF parameters. This method is computationally efficient and suitable for samples with relatively simple surface structures. An adaptive grid search algorithm was developed in this study, and this algorithm utilized a 2-layered grid (step sizes of $1\times 10^{-2}$ and $1\times 10^{-4}$ respectively) structure to incrementally optimize each parameter, providing a more precise approximation of true values. By iteratively narrowing the search range and increasing resolution, this method gradually converges on the optimal solution. The source code of Python for adaptive grid search algorithm was available at https://github.com/PlantSystemsBiology/brdf.

The solver was applied with initial values ($\rho_{0}$, $k_{0}$, $n_{0}$) = (0.5, 0.5, 3) and parameter bounds ρ∈[0,1], k∈[0,1], n∈[1,5] (implemented as [0.01, 0.01, 1.1]–[0.99, 0.99, 5] for numerical stability). Model performance was evaluated by coefficient of determination $\left(R^{2}\right)$, root mean square error (RMSE) and residual analysis, providing reliable estimations of BRDF parameters for each wavelength and leaf sample.

### 2.9 Quantification of leaf roughness using RC

**Fig. 3:**
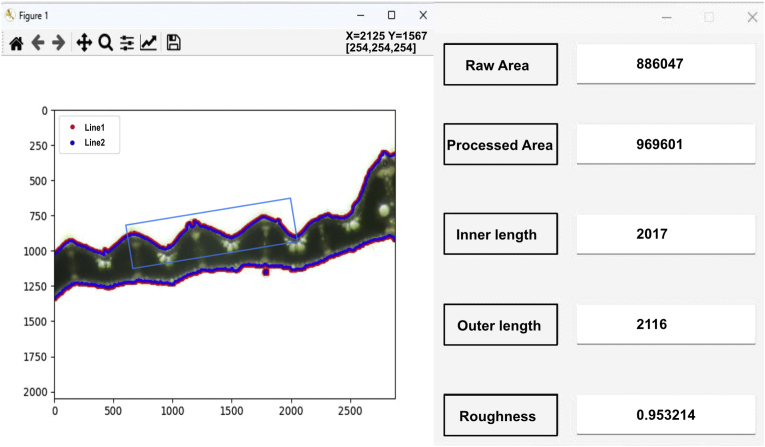
Quantification of leaf surface roughness using the Roughness Calculator (RC) software. The software calculated two edge lengths of the leaf section: the precise inner edge length ($l_{inner}$, blue line) based on individual pixel counts and the smoothed outer edge length ($l_{outer}$, red line) based on a Gaussian smoothing filter. Their ratio (ρ ​= ​*l*_*inner*_*/l*_*outer*_) represented the roughness metric (data were shown at the right side of the software). Manual selection of the region of interest (ROI) (light blue box) allowed analysis for a specific part of leaf surface.

Leaf cross-sections were prepared by manually cutting 50–80 μm segments of fresh leaves with a sharp blade [60], and images of the leaf sections were obtained using an optical microscope (Leica DM2500, Leica Microsystems, Wetzlar, Germany). A custom software tool, Roughness Calculator (RC), was developed to quantify leaf roughness (software available at https://github.com/PlantSystemsBiology/brdf). RC software calculated leaf roughness with the image of leaf cross-section. A region of interest (ROI) of leaf surface was selected from the leaf cross-section image (shown by the blue box) and the exact inner edge length $l_{inner}$ (blue line) based on individual pixel counts was calculated. Then, the smoothed outer edge length $l_{outer}$ (red line) was determined using a Gaussian filter (shown in Fig. 3). The ratio ρ, calculated with Eq. (18), provides a reliable metric for leaf surface roughness. To mitigate the effects of leaf tips and main veins, ROI can be manually selected instead of the whole section within the software. The ROI size was randomized, and five replicates were analyzed for each section image, to minimize user bias. For detailed methodology, see supplementary material (Figs. S4–S8).

(18) $$\begin{array}{c} \rho =\frac{l_{inner}}{l_{outer}} \end{array}$$

### 2.10 Measurement of physiological and biochemical traits

Leaf thickness (T) was measured using a micrometer. Small leaf discs were punched from the leaves avoiding the primary veins for determining the content of chlorophyll *a* (Chl.a), chlorophyll *b* (Chl.b) and carotenoid (Car.), following an established protocol [61]. Absorbance (A) values were measured at wavelengths of 663 ​nm, 645 ​nm and 470 ​nm in a spectrophotometer. The calculation formulas for these pigments were as follows:

(19) $$\begin{array}{c} Chl.a=12.72A_{663}-2.59A_{645} \end{array}$$

(20) $$\begin{array}{c} Chl.b=22.88A_{645}-4.67A_{663} \end{array}$$

(21) $$\begin{array}{c} Car.=\frac{1000A_{470}-3.27C_{chl.a}-104C_{chl.b}}{229} \end{array}$$

For measuring specific leaf weight (SLW), leaf samples with an area of ∼6 ​cm^2^ were collected. The areas of the leaf samples were first precisely measured and then the leaf samples were dried at 105 ​°C for 10 ​min and 80 ​°C until a constant weight. The specific leaf weight (SLW) was calculated by dividing the dry weight of the leaf sample by the area:

(22) $$\begin{array}{c} \text{SLW}=\frac{dry\;weight}{\;area} \end{array}$$

### 2.11 Development of an ensemble learning model for predicting leaf optical properties based on phenotypic traits

In this study, we developed an ensemble learning (EL) model to predict the BRDF parameters of leaves with their phenotypic traits utilizing data from 270 data entries. The EL model integrates Support Vector Regression (SVR), Random Forest Regression (RFR), and Gradient Boosting Regression Tree (GBRT) as base learners, with a stacking strategy using Linear Regression (LR) as the meta-learner (as shown in Fig. 1I).

Nine phenotypic traits were used as input variables, including leaf thickness (T), specific leaf weight (SLW), chlorophyll *a* (Chl.a), chlorophyll *b* (Chl.b), and carotenoid content (Car.) total chlorophyll (Chl.a+b), chlorophyll *a/b* ratio ((Chl.a)/(Chl.b)), leaf surface roughness (ρ), and spectral wavelength (λ). These traits collectively describe the biochemical, physiological, and structural characteristics that determine leaf optical behavior and thus the BRDF parameters.

This EL framework was designed to improve predictive accuracy and generalization by leveraging the complementary strengths of multiple regression algorithms. The methodology involved several key steps.

(1) Data Preprocessing and Standardization

The features were standardized using z-score normalization. For each feature, the mean and standard deviation were calculated, then each sample's feature value was transformed by subtracting the mean and dividing by the standard deviation. This process resulted in a distribution with zero mean and unit variance for all features, eliminating differences in units and scales among features for enhancing model convergence and predictive performance.

(2) Feature Selection

We applied Recursive Feature Elimination (RFE) for feature selection. The RFE iteratively built models and eliminated the least significant features based on feature importance (e.g., model coefficients or feature importance scores). Using this method, five features were selected from the initial set of nine phenotypic traits according to the impact on the BRDF parameters. These five features served as independent variables for model training. The three BRDF parameters were set as target variables, and separate models were developed for each BRDF parameter.

(3) Model Development and Hyperparameter Optimization

The dataset was split into training and testing sets at an 8:2 ratio to ensure robust model training and evaluation. Using the five selected features, we developed three base models: Support Vector Regression (SVR), Random Forest Regression (RFR), and Gradient Boosting Regression (GBRT). For each base model, hyperparameter optimization was performed using 10-fold cross-validation combined with a grid search. By exhaustively testing predefined parameter combinations, a set of parameters that minimized the cross-validation mean squared error (MSE) for each model were identified.

(4) Ensemble Model Construction

We then constructed an ensemble learning (EL) model using a stacking approach. The optimized SVR, RFR, and GBRT models served as primary learners, and Linear Regression (LR) was employed as the secondary learner (meta-learner). This stacking approach allowed the EL model to combine predictions from the primary learners, enhancing the model's overall generalization capability. The performance of the EL model was further evaluated using 10-fold cross-validation.

(5) Model Performance Evaluation

Finally, we evaluated the predictive performance of all models on the test set to verify their generalization abilities and practical applicability. We used Mean Squared Error (MSE) and the coefficient of determination ($R^{2}$) as evaluation metrics to compare the base models and the EL model. This allowed us to assess and compare the accuracy and reliability of each model in predicting the BRDF parameters based on leaf phenotypic traits.

## 3 Results

### 3.1 Ray-tracing analysis of BRDF effects on canopy scattering

To quantify the impact of BRDF parameters on canopy light environments, we incorporated the BRDF parameters in the ray-tracing software previously developed by Song et al. [60] and performed ray tracing simulations using a 3D rice canopy model. The canopy light simulation focused on the distribution of leaf-scattered photosynthetic photon flux density (PPFD), as BRDF parameters mainly regulate the scattering behavior of leaf surfaces rather than atmospheric direct or diffuse light.

**Fig. 4:**
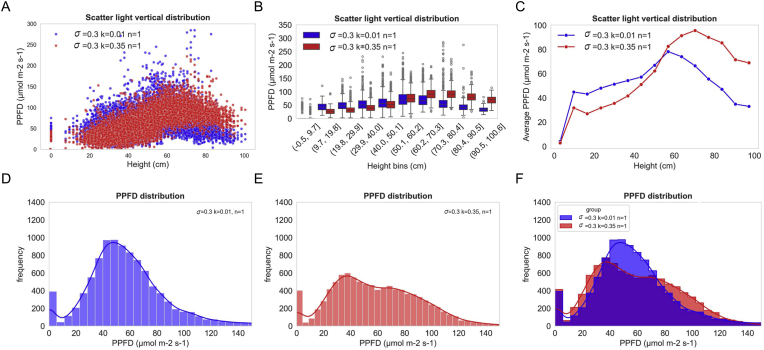
The impact of diffuse reflection coefficient (k) on light distribution within a rice canopy. A: Scatter plot of scattered photosynthetic photon flux density (PPFD) versus canopy height. B: Vertical distribution of scattered PPFD across different canopy layers, presented as box plots. C: Mean scattered PPFD at each canopy layer. D–E: Frequency distribution of scattered PPFD under (D) k ​= ​0.01 and (E) k ​= ​0.35. F: Comparative histogram of scattered PPFD distributions for both scattering coefficients.

When varying the diffuse reflection coefficient (k) between 0.01 and 0.35 while fixing leaf roughness (σ ​= ​0.3) and refractive index (n ​= ​1), marked differences were observed in the spatial patterns of scattered PPFD (Fig. 4A–C). Canopy upper layers exhibited substantially higher scattered PPFD under k ​= ​0.35 compared with k ​= ​0.01. The proportion of leaf facets exposed to medium light intensity (40–80 $\mu mol\;m^{-2}\;s^{-1}$) increased under low k, whereas the fraction under high light intensity (80–150 $\mu mol\;m^{-2}\;s^{-1})$ declined. These findings indicate that a lower diffuse reflection coefficient leads to a more homogeneous scattered light distribution within the canopy.

Further simulations altering σ (0.3 and 1.0) and n (1.0 and 2.2), as well as combined parameter sets (e.g., k ​= ​0.01 with n ​= ​1.0, k ​= ​0.35 with n ​= ​2.2), showed that all three parameters substantially influence canopy-level radiation patterns (Figs. S9–S13). These results emphasize that accurate BRDF parameterization is essential for simulating canopy radiative transfer and photosynthesis.

### 3.2 Variations of leaf anatomical and physiological traits at upper and lower canopy layers in four species

To study the relationship between leaf optical properties and leaf anatomical and physiological traits, we chose four species including two monocotyledonous (maize, rice) and two dicotyledonous (cotton, poplar). The leaves at both upper and lower layers of these plants’ canopies were used for the measurement because the leaves acclimate to heterogeneous light environment in canopy. The phenotypic traits, including leaf thickness (T), specific leaf weight (SLW), chlorophyll *a* (Chl.a), chlorophyll *b* (Chl.b), and carotenoid content (Car.) were quantified. Two-way ANOVA revealed that both species and canopy layer had significant effects (P ​< ​0.001) on T and SLW (Table S3). However, Chl.a, Chl.b, and Car. contents were not significantly affected by either species or layer. Significant species and layer interactions were detected for Chl.a, Chl.b, and Car., indicating that pigment-related traits exhibited species-dependent responses to canopy position.

**Fig. 5:**
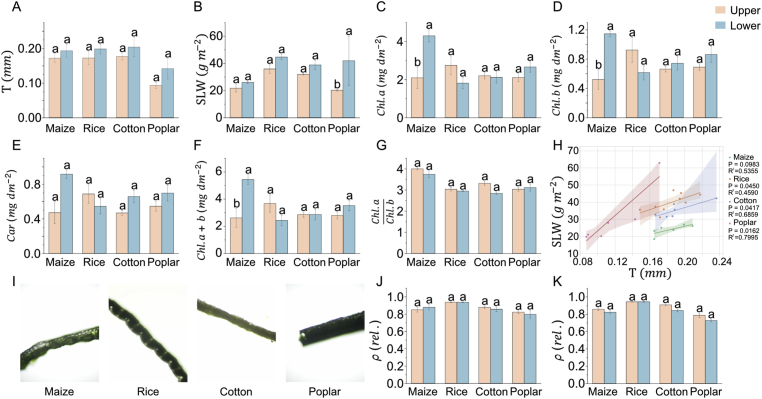
Comparison of anatomical and physiological traits between upper- and lower-layer canopy leaves across four plant species. A–G: leaf thickness, specific leaf weight, chlorophyll *a*, chlorophyll *b*, carotenoid, total chlorophyll and chlorophyll *a*/b ratio in upper (red) and lower (blue) canopy leaves of maize, rice, cotton and poplar. Data were presented as mean ​± ​SE (Standard Error). Significant differences between canopy layers were determined using Two-way ANOVA with Bonferroni multiple post-hoc tests, marked with ∗ for P<0,05. H: linear regression between specific leaf weight and thickness across four species, with 95 ​% confidence intervals. The regression lines and coefficient of determination ($R^{2}$) values were provided for each species. I: leaf section microscopy image examples. J–K: leaf surface roughness for the adaxial side (J) and abaxial side (K).

The leaf thickness was not significantly different between upper and lower canopy layers across species (Fig. 5A). SLW was significantly higher in lower-layer poplar leaves compared to upper-layer leaves (P<0.05) (Fig. 5B), possibly reflecting structural adaptations to lower light levels in the shaded canopy [62]. In contrast, SLW differences between canopy layers were less pronounced in maize, rice, and cotton. A positive correlation between SLW and T across species were observed, with poplar exhibiting the strongest correlation ($R^{2}>0.79$) and rice the weakest ($R^{2}>0.45$) (Fig. 5H). The relationship between SLW and T was dramatically different among these four species, shown by the shaded areas representing the 95 ​% confidence intervals, highlighting interspecies variation in leaf structure and density. We also calculated total chlorophyll (Chl.a+b) and the ratio of chlorophyll *a* to chlorophyll *b* (Chl.a/Chl.b). In maize, the chlorophyll content (Chl.a and Chl.b) was significantly higher in lower-layer leaves (Fig. 5C–D, F), but rice exhibited the opposite pattern. As shown in Fig. 5G, the chlorophyll *a*/b ratio was not different between canopy layers. In maize, the *Car.* in lower-layer leaves nearly doubled that in upper-layer leaves (Fig. 5E). We also quantified the leaf surface roughness (ρ) based on the leaf section images using the developed Roughness Calculator (RC) software (Fig. 5I). The results indicate that the position of the leaf within the canopy has no significant effect on the ρ of the leaf, with minimal differences between the adaxial (Fig. 5J) and abaxial (Fig. 5K) surfaces and consistent numerical trends. Rice leaves are the roughest, while poplar leaves are the smoothest.

### 3.3 Spatial distribution of reflectance across species and canopy layers

**Fig. 6:**
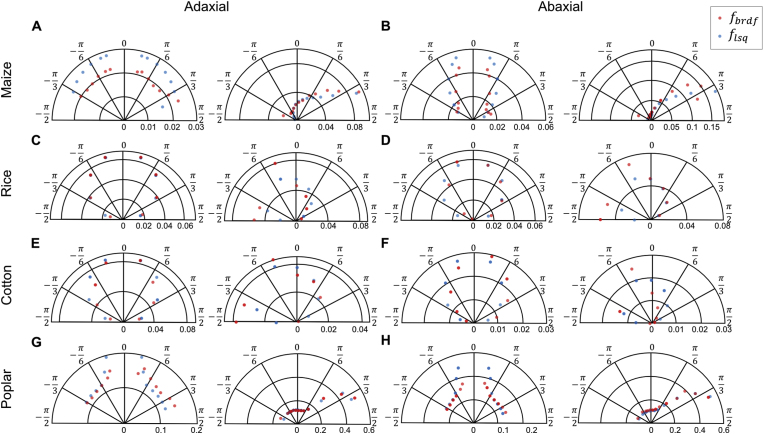
Reflectance distribution for the adaxial and abaxial surfaces of four species in polar coordinate. The reflectance distributions for the adaxial (A, C, E, G) and abaxial (B, D, F, H) surfaces of leaves across four plant species: maize (A–B), rice (C–D), cotton (E–F), and poplar (G–H). For each figure panel, data of two illuminated angles (light incident angles) were shown. For maize and poplar, 0 (left) and 11π/36 (right) were shown, and for rice and cotton, 0 (left) and π/4 (right) were shown. In the polar coordinate, the angle represents the reflection angle, the radial represents the value of the BRDF, with the pink asterisk representing the actual measured reflectance ($f_{brdf}$) by the DSDI system and the green circle representing the fitted reflectance ($f_{lsq}$) on the BRDF model by the least squares curve fit. Units of reflectance are given in $sr^{-1}$.

Using the DSDI system developed in this study, we performed the measurement of BRDF for the samples used for quantifying those anatomical and physiological traits above. Both adaxial and abaxial surfaces of the leaves were measured. Then, the Cook Torrance BRDF model was used to fit the data for deriving the BRDF parameters (Fig. 6). The reflectance distribution on leaf surfaces generally consists of a narrow peak (e.g., the blue ellipse in Fig. 1B) in the specular reflection direction, superimposed on a more uniform diffuse background (e.g., the red semicircle in Fig. 1B) in the diffuse reflection directions [37]. The maximum specular peak, indicative of nearly pure specular reflection, reaches approximately 0.6 $sr^{-1}$ within a small solid angle for poplar leaves at light incident angle 11π/36 (Fig. 6). Notably, rice and cotton exhibit relatively uniform reflectance distributions across both adaxial and abaxial surfaces, with lower reflectance values compared to maize and poplar. This pattern may arise from specific structural or biochemical properties in rice and cotton leaves that reduce directional reflection.

Interestingly, the peak of the BRDF appears at an angle slightly larger than the ideal specular reflection angle, an effect influenced by the σ(λ) [55]. Although the diffuse reflection component appears minor, it dominates when integrated over the entire hemisphere [37]. As the zenith illumination angle increases, all samples become more specular, with narrower and more pronounced BRDF peaks. Despite these common trends, the four species exhibit distinct BRDF profiles, allowing clear differentiation based on reflectance distribution patterns.

The polar plots in Fig. 6 visualize these variations in reflectance distribution across species. Maize and poplar display pronounced specular reflections, particularly on the abaxial surfaces (Fig. 6A–B, G-H), while the BRDF peaks of rice and cotton (≈0.08 sr^−1^) were significantly lower than those of maize (0.15 sr^−1^) and poplar (0.6 sr^−1^) (P ​< ​0.01). (Fig. 6C–F). These findings underscore the importance of species-specific leaf surface adaptations in controlling light distribution within the canopy, with potential implications for enhancing photosynthetic efficiency. Such adaptations provide critical insights for refining light interaction models and highlight potential targets in breeding programs aiming at improving resource efficiency and productivity in diverse light environments.

### 3.4 Evaluation of BRDF parameter fitting

**Fig. 7:**
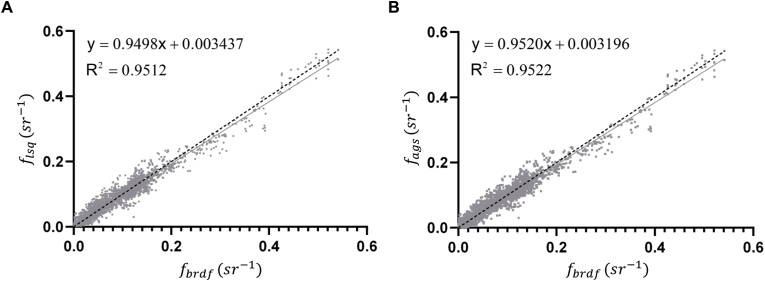
Comparison between measured and fitted BRDF values using two fitting methods, Least Squares curve fit function (A) and the Adaptive Grid Search (B). *f*_*lsq*_ indicates BRDF values simulated using the Least Squares fitting method, and *f*_*ags*_ denotes values derived from the Adaptive Grid Search method. The black dotted line represents the 1:1 line, while the solid regression lines illustrate the relationship between fitted bidirectional reflectance (*f*_*lsq*_ and *f*_*ags*_) and measured bidirectional reflectance ($f_{brdf}$). The coefficient of determination ($R^{2}$) indicates the strength of correlation, with both methods showing strong agreement with the measured data ($R^{2}>\;0.95$). The scatter points present the bidirectional reflectance for various combinations of incident angles and reflection angles.

To evaluate the performance of the BRDF parameter fitting, we applied two methods ($f_{lsq}$ for Least Squares and $f_{ags}$ for Adaptive Grid Search) to fit the BRDF, based on the actual measured reflectance of both leaf surfaces for upper and lower layer in canopies of the four species. The fitted reflectance values ($f_{lsq}$ and $f_{ags}$) were linear correlated ($R^{2}$ ​= ​0.9512 and 0.9522) with the actual measured reflectance values ($f_{brdf}$) (Fig. 7) at the measurement locations. Both methods provided high-quality fits to the measured data ($R^{2}>\;0.95$), effectively capturing the reflectance distribution on leaf surfaces. While the Adaptive Grid Search achieved slightly higher fitting accuracy due to its two-layer grid structure, but it required longer processing times. Therefore, we used the results from the Least Squares fitting method for further analysis.

### 3.5 Variations of BRDF parameters across species and canopy layers

**Fig. 8:**
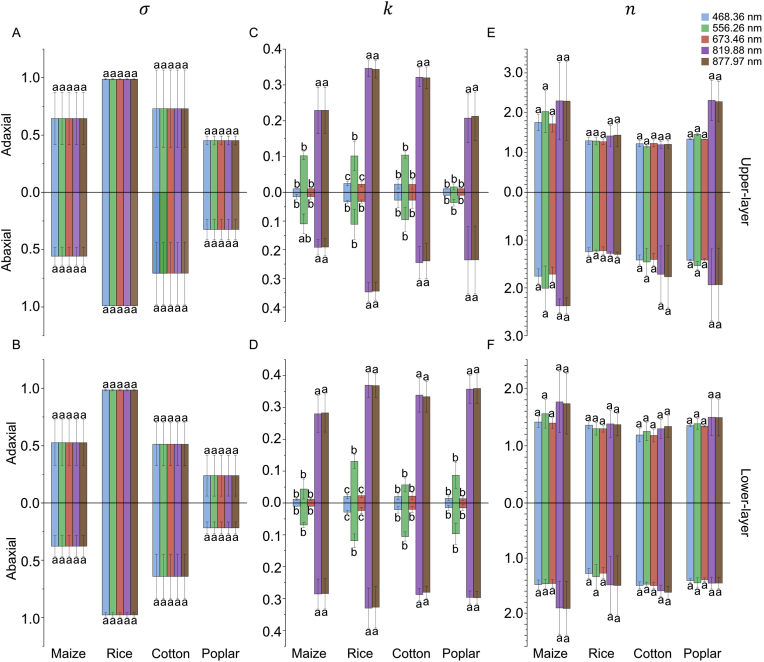
Comparison of BRDF parameters across species (maize, rice, cotton, and poplar) and canopy layers (upper and lower) for both adaxial and abaxial leaf surfaces. These parameters included roughness σ(λ) (A–B), diffuse reflectance coefficient k(λ) (C–D) and refractive index n(λ) (E–F). Each parameter was measured at multiple wavelengths (468.36 nm, 556.26 nm, 673.46 nm, 819.88 nm and 877.97 nm), represented by different colors. Data were presented as mean ​± ​SE (Standard Error) for upper-layer (A, C, E) and lower-layer (B, D, F) leaves. Mean comparisons were conducted using Tukey's HSD test at a significance level of 0.05. Different letters indicate significant differences among different wavelengths.

There were significant variations for BRDF parameters across species, canopy layers and light wavelength (Fig. 8). Rice and cotton exhibited higher σ(λ) values than that of maize and poplar for both two leaf surfaces and two canopy layers, indicating that the rice and cotton had irregular surface structure compared to maize and poplar. This higher roughness results in a relatively even reflectance distribution (Fig. 6C–F). In contrast, maize and poplar show higher specular reflectance components (Fig. 6A–B, G-H) due to their lower σ(λ) values, which increase the probability of concentrated light reflection [63]. The σ(λ) was the same for different wavelengths, suggesting that this parameter was a property of leaf surface texture controlled by species-specific structural traits [64]. In contrast, the k(λ) demonstrates marked sensitivity to wavelength, with the highest values at infrared region and relatively high at green light and lower for blue and red light (Fig. 8C–D). This phenomenon can be explained by the reflectance of leaf at these wavelengths. The wavelength-dependent nature of k(λ) aligns with general spectral reflectance properties, where longer wavelengths tend to exhibit more diffuse scattering [51]. The n(λ) shows limited wavelength sensitivity across species, with relatively high values in maize and poplar (Fig. 8E–F). Notably, n(λ) remains consistent between adaxial and abaxial surfaces, supported by the similar structure of leaf surface at both side of a leaf.

### 3.6 Correlation analysis between leaf phenotypic traits and BRDF parameters

**Fig. 9:**
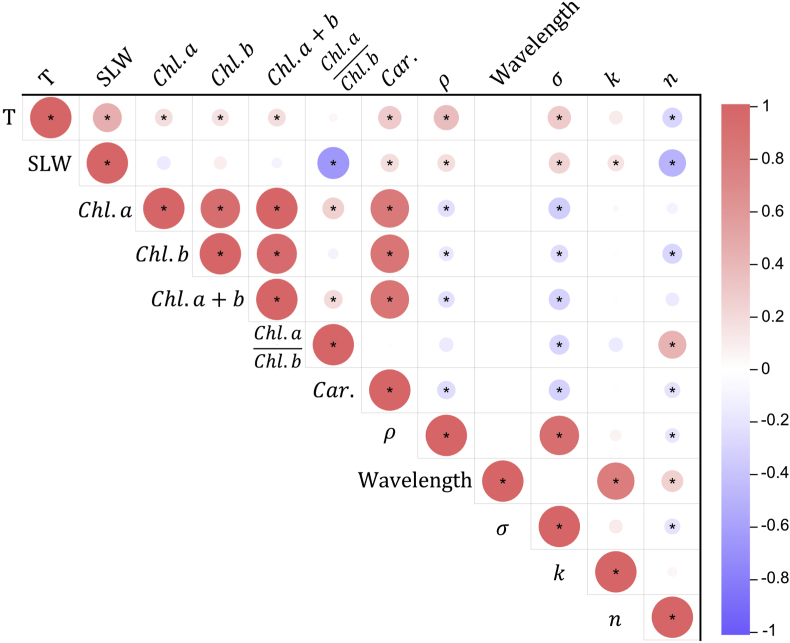
Correlation matrix between wavelengths, leaf traits and BRDF parameters. This heatmap shows the correlation coefficients among various parameters, including Leaf thickness (T), Specific leaf weight (SLW), Chlorophyll *a* (Chl.a), Chlorophyll *b* (Chl.b), Carotenoid content (Car.), Total chlorophyll (Chl.a+b), Ratio of chlorophyll *a* to b ((Chl.a)/(Chl.b)), leaf surface roughness (ρ), measurement wavelength (λ), and the BRDF parameters σ(λ), k(λ), and n(λ). The wavelength feature represents the spectral band (400–992 ​nm) at which BRDF parameters were measured. The color scale represents the strength and direction of correlations, with red indicating positive correlations and blue indicating negative correlations. The color intensity and size of the circle correspond to the strength of the correlation, as shown in the color bar. Pearson's correlation coefficient was used, and significant correlations were marked with an asterisk (∗) at the P<0.05 level.

To further understand the relationship between leaf phenotypic traits and optical properties, we performed a correlation analysis between relevant parameters, as presented in Fig. 9. The analysis reveals a highly significant positive correlation between σ(λ) and the leaf surface roughness ρ, with no significant correlation between σ(λ) and wavelength. This finding suggests that σ(λ) primarily reflects the intrinsic surface characteristics of the leaf, which can be reliably modeled through a linear relationship based on ρ (Fig. S8). Additionally, σ(λ) shows a significant negative correlation with pigment content.

The k(λ) was strong positive correlated with λ, consistent with known spectral reflectance patterns in leaves, where light scattering generally increases with longer wavelengths (from visible light to infrared light) [65]. It should be noted that k(λ) was indeed correlated with the leaf absorbance for specific band of wavelength.

The n(λ) exhibits significant negative correlations with both T and SLW. This indicates that denser and thicker leaves, which associated with higher SLW and T, tend to have lower refractive indices, thereby reducing light transmittance. Furthermore, there is a noteworthy negative correlation between σ(λ) and n(λ), suggesting that as surface roughness increases, the refractive index decreases. This relationship implies that the leaf surface structure may be biologically connected to its internal composition, potentially affecting its refractive properties [66].

### 3.7 Predictive model for BRDF parameters with data of leaf anatomical and physiological traits

**Fig. 10:**
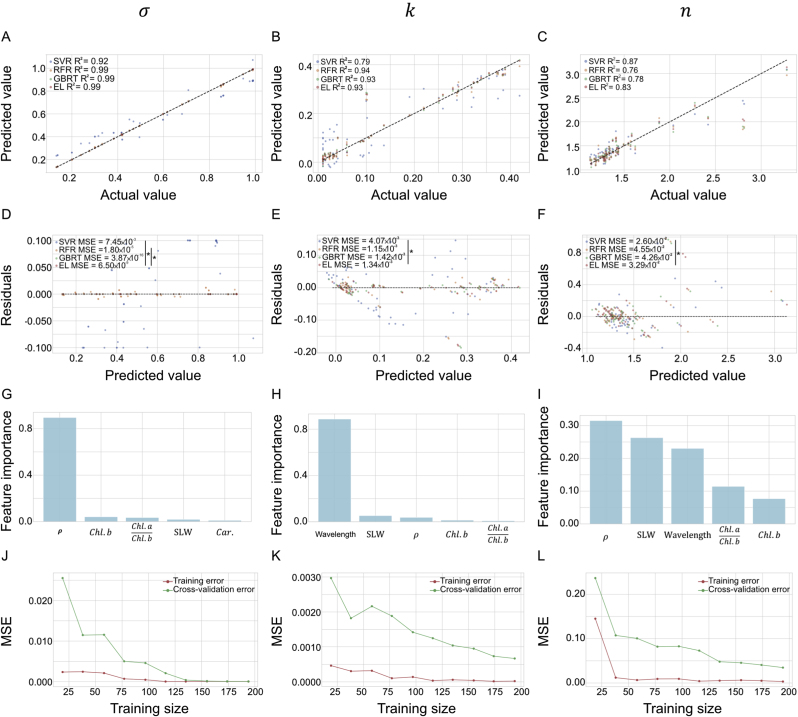
Performance of ensemble learning (EL) model for predicting BRDF parameters σ(λ), k(λ), and n(λ) based on leaf traits. A–C: the scatter plots of actual versus predicted values for σ(λ), k(λ), and n(λ), respectively, with a dotted line indicating the 1:1 relationship. D–F: the residual plots for each parameter, illustrating the residual distribution against predicted values. G–I: Importance of leaf phenotypic traits for predicting BRDF parameters in RFR model. J–L: present the learning curves, showing both training error (red line) and cross-validation error (green line) for the EL model during the training process for each parameter. Model comparisons were based on SVR (Support Vector Regressor), RFR (Random Forest Regressor), GBRT (Gradient Boosting Regressor), and a stacking EL model. The statistical metrics (MSE and $R^{2}$) indicate the accuracy and robustness of the models, with the stacking model showing superior performance for most parameters. Statistical significance was assessed via paired t-tests, with a significance level of P<0.05, denoted by an asterisk (∗).

To predict the BRDF parameters using the data of leaf anatomical and physiological traits, several base models and the ensemble learning model were trained and evaluated. Results show that the R^2^ of stacking ensemble learning (EL) model was generally higher than individual base models, including Support Vector Regression (SVR), Random Forest Regression (RFR), and Gradient Boosting Regression Tree (GBRT), in predicting BRDF parameters (σ(λ), k(λ), and n(λ)). The comparison of models shows that the EL model consistently outperforms the individual models, yielding $R^{2}$ value 0.83–0.99 on the test set (Fig. 10A–C). However, all models demonstrated a relatively lower performance in predicting n(λ), likely due to additional influencing factors, such as water content, which affect light transmission properties in leaves [26].

To assess statistical significance in model performance, paired t-tests were conducted. Fig. 10D–F reveals that the EL model had a significantly lower Mean Squared Error (MSE) than the SVR model (P<0.05), though differences between the EL model and RFR or GBRT were not statistically significant. Feature importance analysis (Fig. 10G–I) indicated that leaf surface roughness (ρ) and wavelength (λ) were the dominant predictors for BRDF parameters σ(λ), k(λ), and n(λ), followed by specific leaf weight (SLW) and pigment-related traits ((Chl.a)/(Chl.b) and Chl.b). These results suggest that both structural and spectral features play critical roles in determining leaf optical features. The learning curves (Fig. 10J-L) show the training and cross-validation error for each model, with the EL model achieving lower cross-validation error, indicating improved generalization capability for unseen data.

These findings confirm that the stacking EL model provides robust and accurate predictions for BRDF parameters by effectively integrating the strengths of each base model. The enhanced predictive power of the EL model offers a reliable framework for modeling leaf optical properties based on leaf traits, which could be instrumental in refining canopy light distribution models and optimizing crop canopy.

## 4 Discussion

This study introduces an integrated framework combining optical instrumentation (DSDI), physics-based modeling (BRDF), and data-driven analytics (EL) to quantify and predict leaf optical properties from anatomical and biochemical traits. Unlike traditional optical methods limited to slow reflectance or transmittance measurements, the proposed approach transforms optical characterization into a phenotype-driven, scalable, and computationally extensible process. The DSDI system enables precise measurement of directional reflectance across multiple angles, and the derived BRDF parameters (σ, k, and n) capture critical variations in surface roughness, scattering, and refractive properties that define leaf optical diversity among species and canopy layers.

### 4.1 Integration of optical traits into phenomics frameworks

Three-dimensional (3D) canopy photosynthesis models can be used to identify factors controlling canopy photosynthesis efficiency and deconvolute dominant factors governing canopy photosynthetic performance [[67], [68], [69], [70]]. The distribution of direct light and diffuse light has been found to have a significant impact on the photosynthetic efficiency of the canopy [71]. Plant architectural traits, such as leaf type and leaf angle, also influences light distribution in plant canopy.

One of the major predictions of such canopy photosynthesis models is that canopy with vertical light-green leaves in upper canopy coupled with horizontal dark-green foliar arrangements in lower canopy can help increase efficiency [63]. Such a canopy architecture can enhance photon flux homogeneity, simultaneously mitigating light saturation in apical leaves while alleviating light limitations in basal leaves. In this study, leaf anatomical and biochemical features are major determinants of optical properties of the leaf [72,73]. Our results reveal a statistically robust correlation between the roughness parameter (σ(λ)) of the BRDF model and leaf surface roughness (ρ) derived from leaf cross-sectional microscopy quantifications (Fig. 9). The established linear regression model (Fig. S8) enables practical estimation of σ(λ) through rapid ρ characterization using microscopy-based measurements. This positive correlation suggests that leaf surface irregularities directly influence BRDF roughness, likely enhancing light scattering within the canopy. A pronounced correlation was observed between the diffuse reflection coefficient (k(λ)) and wavelength (Fig. 9). This relationship aligns with the known wavelength dependence of leaf spectral reflectance [74]. The refractive index (n(λ)) demonstrated a negative correlation with leaf thickness and specific leaf weight (Fig. 9). This finding suggests that thinner leaves in the upper layer tend to transmit more light, which could be advantageous in light distribution and utilization for the whole canopy, particularly for species such as maize and poplar [75]. These new observations provide a new dimension of crop canopy engineering, i.e. manipulating the leaf optical properties instead of canopy architectures for a better light environment inside a canopy for greater efficiency.

### 4.2 Effectiveness of the EL model for predicting optical properties

Our ensemble learning (EL) model effectively predicted leaf optical properties (σ(λ), k(λ) and n(λ)) based on phenotypic traits, with high accuracy ($R^{2}>0.83$ for all parameters, as shown in Fig. 10). However, the relative weaker predictive power for n(λ) ($R^{2}=0.83$) suggests unaccounted factors, such as leaf water content [26] or other tissues [54], influence the leaf refractive index. The dominance of ρ highlights the critical role of epidermal microstructure in controlling the roughness parameter σ(λ), consistent with previous studies linking surface topology to directional scattering. The significant contribution of λ reflects the wavelength-dependent variation in pigment absorption and scattering, affecting both the diffuse reflection coefficient k(λ) and refractive index n(λ). Meanwhile, SLW and chlorophyll-related traits contributed secondarily, indicating that leaf thickness and pigment composition modulate internal light transmission and absorption. Together, these findings demonstrate that the EL model not only achieved accurate prediction but also captured biologically interpretable relationships between leaf structure, pigment composition, and BRDF parameters.

### 4.3 Biological relevance and future directions

Our findings underscore that leaf optical properties represent an important yet underutilized dimension of plant phenotyping. The correlations between BRDF parameters and phenotypic traits such as leaf thickness, pigment composition, and surface microstructure highlight the potential of optical phenotyping to reveal functional adaptations of plant leaves. By integrating BRDF-predicted parameters into 3D canopy photosynthesis models, this framework enables virtual experiments for testing how structural or biochemical modifications affect canopy-scale light distribution, providing a foundation for data-driven crop ideotype design. Furthermore, when integrated with 3D point cloud data acquired from phenotyping platforms, the proposed method allows indirect scalable evaluation of canopy light distribution and photosynthetic efficiency, supporting computational phenotyping of canopy photosynthesis.

As the current dataset covers four representative species across two canopy positions, this limited biological diversity constrains the generalizability of the predictive model. The present study primarily focuses on developing an integrated hardware–software framework for predicting leaf optical properties from structural and biochemical traits, rather than achieving exhaustive species coverage. The selected species were chosen to represent both monocotyledonous and dicotyledonous groups, ensuring methodological diversity rather than taxonomic completeness. It is noteworthy that the prediction model for certain optical parameter, such as surface roughness (σ), could be relatively conserved across species because they primarily depend on epidermal microstructure. In contrast, models for predicting parameters like the diffuse reflection coefficient (k) and refractive index (n) might exhibit species- and condition-specific variability, driven by biochemical composition and internal tissue organization.

Future studies should expand this framework across genotypes, environmental conditions, and stress treatments to construct universal predictive models for leaf optical behavior. The combination of optical phenotyping, machine learning, and radiative transfer modeling represents a promising direction for next-generation plant phenomics, where digital and computational tools jointly enable predictive understanding of plant function and light-use efficiency.

## 5 Conclusion

This study establishes an integrated framework that bridges optical physics and plant phenomics by combining a custom-designed Directional Spectrum Detection Instrument (DSDI), Bidirectional Reflectance Distribution Function (BRDF) modeling, and ensemble learning (EL) model. The framework enables accurate and scalable quantification of leaf optical properties and provides the predictive relationship between BRDF parameters and leaf phenotypic traits across multiple species. The DSDI system precisely measures directional reflectance, while roughness (σ), diffuse reflection coefficient (k), and refractive index (n), capture key mechanisms governing leaf light scattering and absorption. The EL model achieved high prediction accuracy (R^2^ ​= ​0.83–0.99), demonstrating that leaf structural and biochemical traits can reliably predict optical behavior. Ray-tracing simulations further confirmed that BRDF parameters strongly influence canopy light distribution, highlighting their importance for parameterizing 3D canopy photosynthesis models. Overall, this work advances phenomics-oriented optical characterization by linking measurable leaf traits to canopy-scale light modeling, providing a scalable, data-driven pathway toward predictive phenotyping and digital crop design for improved photosynthetic efficiency.

## Author contributions

X.Z. and Q.S. designed and conceived the study. Q.S. developed the equipment and ray tracing software. Q.S., L.M., and Y.W. performed the experiments. L.X.Y., X.G., and M.W. developed software for image analysis, L.D. and Q.S. analyzed the data, built models and wrote the paper with the inputs from all authors. Y.Z., Q.S., and X.Z. supervised the study.

## Funding

We acknowledge the financial support of the 10.13039/501100001809National Natural Science Foundation of China (32270428, U22A20464) to Q.S. and X. Z., the Earmarked Fund for XJARS-Cotton (XJARS-03) to Y.Z., Open Project of the Key Laboratory of Oasis Eco-agriculture, 10.13039/501100009967Xinjiang Production and Construction Corps (202102) to Q.S., the Strategic Priority Research Program of the 10.13039/501100002367Chinese Academy of Sciences (XDB0630300) and CAS-NWO Joint grant (482.20.700) to Q. S. and X. Z., the Natural Science Foundation of Shanghai (23ZR1455900) and the 10.13039/501100013105Shanghai Rising-Star Program (21QC1401200) to Q. S.

## Declaration of interest statement

The authors declare that they have no known competing financial interests or personal relationships that could have appeared to influence the work reported in this paper.

## Data Availability

The source code used in this study is available for noncommercial use and the code can be downloaded from https://github.com/PlantSystemsBiology/brdf. The updated ray tracing in this study is available for noncommercial use and the code can be downloaded from https://github.com/PlantSystemsBiology/fastTracerPublic. The data of this study are available from the corresponding author upon request.
