# Supplementary material for: Leaf bidirectional reflectance distribution function (BRDF) prediction with phenotypic traits in four species: Development of a novel measuring and analyzing framework
Source: Plant Phenomics. 2025 Oct 30;7(4):100135. doi: 10.1016/j.plaphe.2025.100135 (PMC13109308; doi:10.1016/j.plaphe.2025.100135)
Supplement: Multimedia component 1 [file mmc1.docx]

**Supplementary Information**

**This file includes additional descriptions, tables, and figures supporting the manuscript entitled:**

**“Leaf Bidirectional Reflectance Distribution Function (BRDF) Prediction with Phenotypic Traits in Four Species: Development of a Novel Measuring and Analyzing Framework”**

**Liangchao Deng^1,2^, Leo Xinqi Yu^2^, Linxiong Mao^2^, Yanjie Wang^2^, Xiyue Guo^3^, Minjuan Wang^3^, Yali Zhang^1^*, Qingfeng Song^2^*, Xin-Guang Zhu^2^***

All supplementary texts, tables, and figures are sequentially numbered and referenced in the main manuscript.

**Description and verification of the Directional Spectrum Detection Instrument (DSDI)**

The Directional Spectrum Detection Instrument (DSDI) is an optical System specifically designed for measuring the directional distribution of transmitted and reflected light in spatial. By leveraging mechanical rotation of the ring and shaft, the relative motion between the silicon photodetector and the leaf allows direct measurement of the reflectance or transmission light. The light source, a halogen lamp, operates across a wavelength range of $199.49-992.43nm$. Each of the left and right two arc slides respectively have a receiving probe sliding along the arc. The zenith angle of the receiver is changed by accepting the relative sliding of the probe on the arc. The incidence angle is changed by the axial rotation of the sample holder. By rotating the arc around the spindle, the azimuth angle of the detector can be changed. Furthermore, the reflected light and transmitted light in different directions can be obtained. Due to the mechanical defects of the spectral goniometer, the base bracket hinders the sliding of the receiving probe, which leads to the inability to measure the light intensity at the zenith angles of $-\frac{\pi}{36}\sim\frac{\pi}{36}$ and $\frac{35\pi}{36}\sim\frac{37\pi}{36}$.


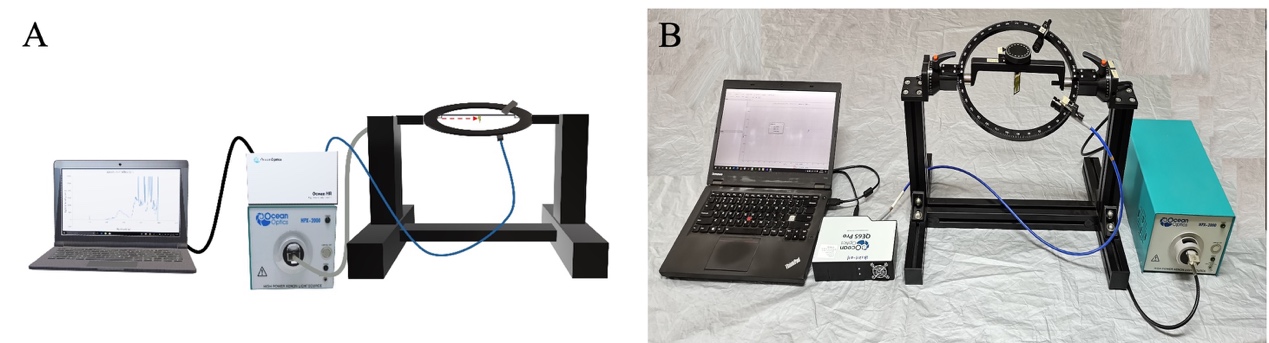


**Figure S1. Schematic diagram and photograph of the custom-built Directional Spectrum Detection Instrument (DSDI).** A: CAD-based schematic model showing the structural design and optical configuration of the DSDI system. B: Photograph of the actual instrument used in this study, consisting of the fiber spectrometer (Ocean Optics, USA), rotation platform, and control computer.

Principle

The DSDI system is composed of several key components, including the HPX-2000 high-power xenon light source (Ocean Insight Inc., USA), the HR2000 high-resolution fiber optic spectrometer (Ocean Insight Inc., USA), sample scaffold and PC. The HPX-2000 provides a broad, continuous spectrum of light ranging from UV to the NIR. The HR2000 is responsive to wavelengths ranging from 200 to 1100 $\mathrm{nm}$, with a high optical resolution of 0.065 $\mathrm{nm}$ (The simple specifications of HPX-2000 and HR-2000 are shown in Table S2and Table S3 below.). The sample scaffold consists of a sample holder that can rotate vertically and a ring that can rotate around the spindle, providing a range of detection angles. Two spectral detection probes are placed on either side of the ring, while a light source probe can be placed at one end of the spindle. The PC is used to install OOIBase32 software, which is capable of reading data from the spectrometer (For more detailed specifications of the HPX-2000 and HR2000 components, please refer to attachments 1 and 2 in the folder.).

**Table S1. HPX-2000 high-power xenon light source specifications.**

| Specifications |  |
| --- | --- |
| Dimensions: | 145mm×165mm×260mm |
| Weight: | 5kg |
| Power consumption: | 60 W AC |
| Wavelength range: | 185-2000nm |

**Table S2. HR-2000 high-resolution fiber optic spectrometer specifications.**

| Specifications |  |
| --- | --- |
| Dimensions: | 148.6mm×104.8mm×45.1mm |
| Weight: | 570g |
| Power consumption: | 90 mA@ 5 VDC |
| Wavelength range: | 200-1000nm |

Detecting the distribution of reflected light is essentially detecting the light in each angular direction on the reflected semicircle. Based on the principle of mechanical rotation of the ring and the shaft, then directly measuring the distribution of the reflected or transmitted light radiant flux of the object by using the relative motion between the silicon photodetector and the measured object.

Each of the left and right two arc slides respectively have a receiving probe sliding along the arc. The zenith angle of the receiver is changed by accepting the relative sliding of the probe on the arc. The incidence angle is changed by the axial rotation of the sample holder.

# **Process**

The measurement procedure using DSDI is as follows:

1. Select the appropriate position of the sample to be tested, adjust the leaf holder placing the position to be tested in the middle of the sample holder, and adjusting the surface of sample to be tested is facing the light source.
2. Rotate the leaf holder to adjust the zenith angle of the incident light.
3. Turn on the light source, rotate the ring to adjust the azimuth angle of the detector and move the collimation lens along the ring to adjust its zenith angle.
4. Read the spectral reflection curve of each measurement in the OOIBase32 installed on the PC.

# **Verification**

To verify the accuracy of DSDI, we performed a test using a neutral standard whiteboard without characteristic absorption as a reference surface. We measured the reflectance distribution at zenith angles of $0^{\circ}$, $30^{\circ}$, and $45^{\circ}$ respectively in the main plane. The results are shown in Figure S9.


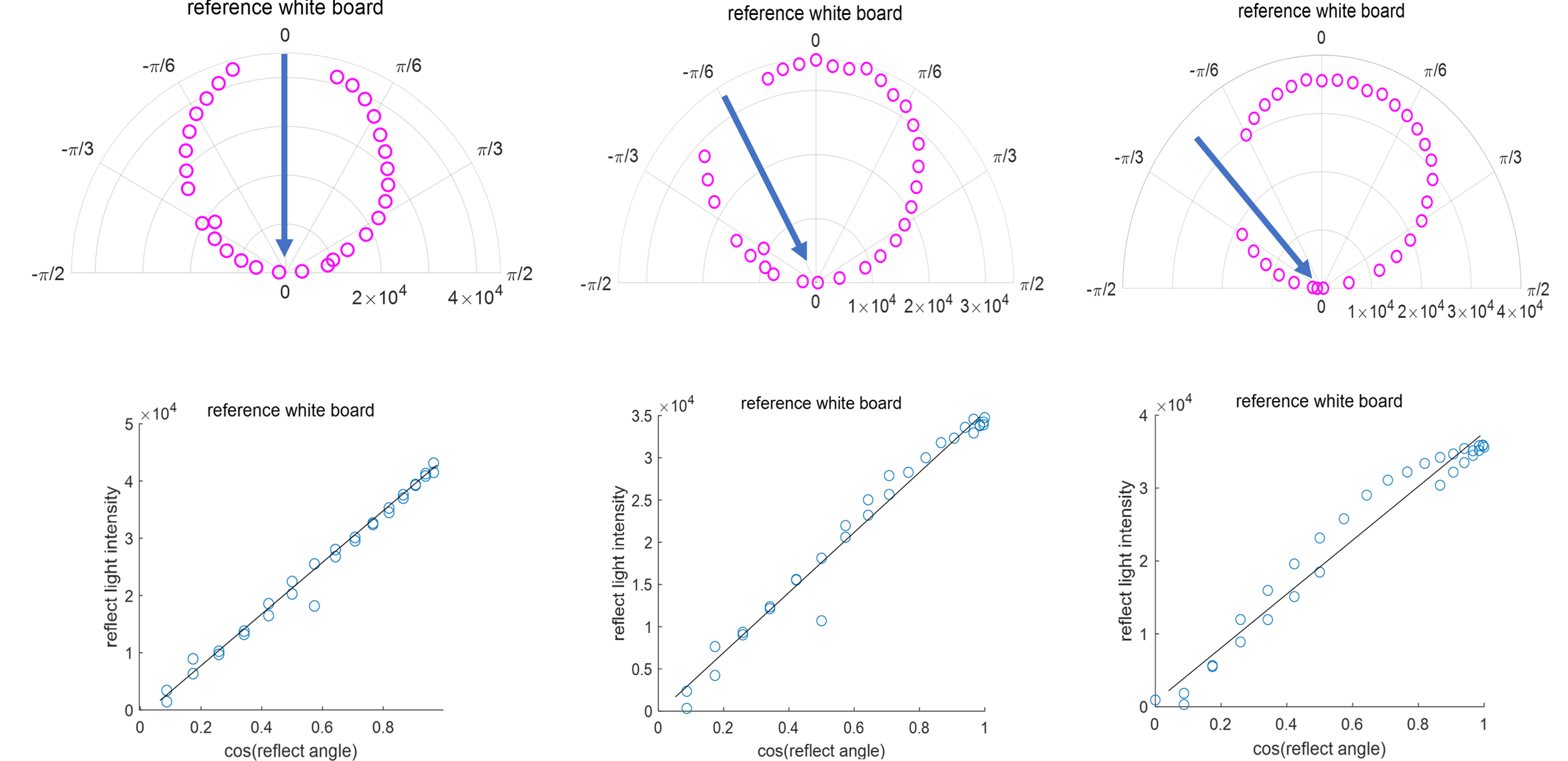


**Figure S2. The reflect light intensity of standard white board at different zenith angles.**

The reflectance of a whiteboard is typically assumed to approximate the Lambertian reflectance model, which states that the radiance of each reflection direction is constant. As a result, there should be a linear relationship between the radiation intensity and the cosine value of the detection angle. Figure S5 shows that the results of the DSDI measurements are consistent with this characteristic, indicating that DSDI can accurately measure the spatial light distribution of objects.


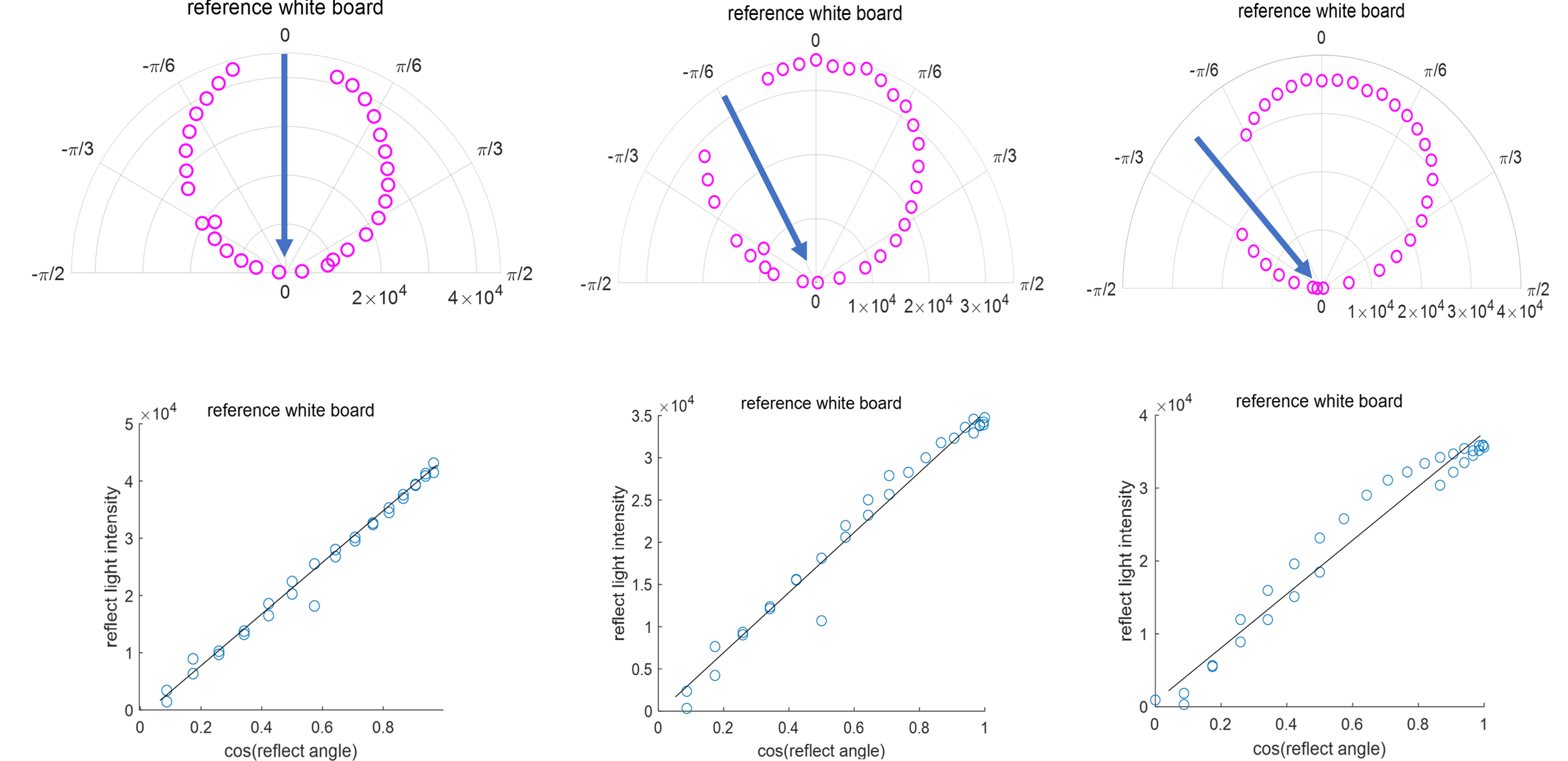


**Figure S3. The relationship between the reflect light intensity of standard white-board and** $\boldsymbol{cos(\theta)}$**.** $\theta$ is the zenith angle between detection direction and incident direction.

# **Conclusion**

The primary objective of DSDI is to measure the directional distribution of transmitted and reflected light in spatial. The experimental results show that the measurement results are in good agreement with the actual results. The DSDI can be used as a tool to measure the directional distribution of transmitted and reflected light in spatial. It provides the convenient tools for the accurate measurement of light distribution of leaves. Compared to the stepper motor shaft, its advantage is that the angle can be measured more.

**Description and verification of the Roughness Calculator (RC)**

RC (roughness calculator) is an open-source leaf surface roughness calculation software based on python. RC aims to provide a way to quantify surface roughness parameters and accelerate the use of BRDF models in simulating leaf light distribution. As an open-source software, RC allows other users to modify code based on the original code. The software and code are available at <https://github.com/PlantSystemsBiology/brdf>.

# **Principle**

Here, we will briefly introduce the principle of RC. The first step is to create an x-y coordinate system and import the section image. In the second step, the image is denoised using the binarization method to obtain a clean image. The third step involves converting the image into a grayscale format to obtain the edge of the leaf section and the edge after Gaussian filtering. In the fourth step, the area and perimeter are calculated based on the edge of the leaf section. The fifth step involves using the lasso tool to obtain the intersection of the drawing area and the edge of section, enabling the determination of the length of the region of interest. Then the roughness is calculated in the sixth step using a formula.

# **Process**

In order to facilitate the use, we designed a visual interface and compiled python code into RC. The interface of RC is shown in Figure S6. The interface of RC consists of the following components:

- The software logo and name are located in the upper left-hand corner of the interface.
- The center of the left side of the interface displays the image processing area.
- The three buttons located in a row beneath the left side of the interface provide access to the functional areas, which include "upload," "calculate," and "lasso select."
- The right side of the interface displays the results of the image calculations.


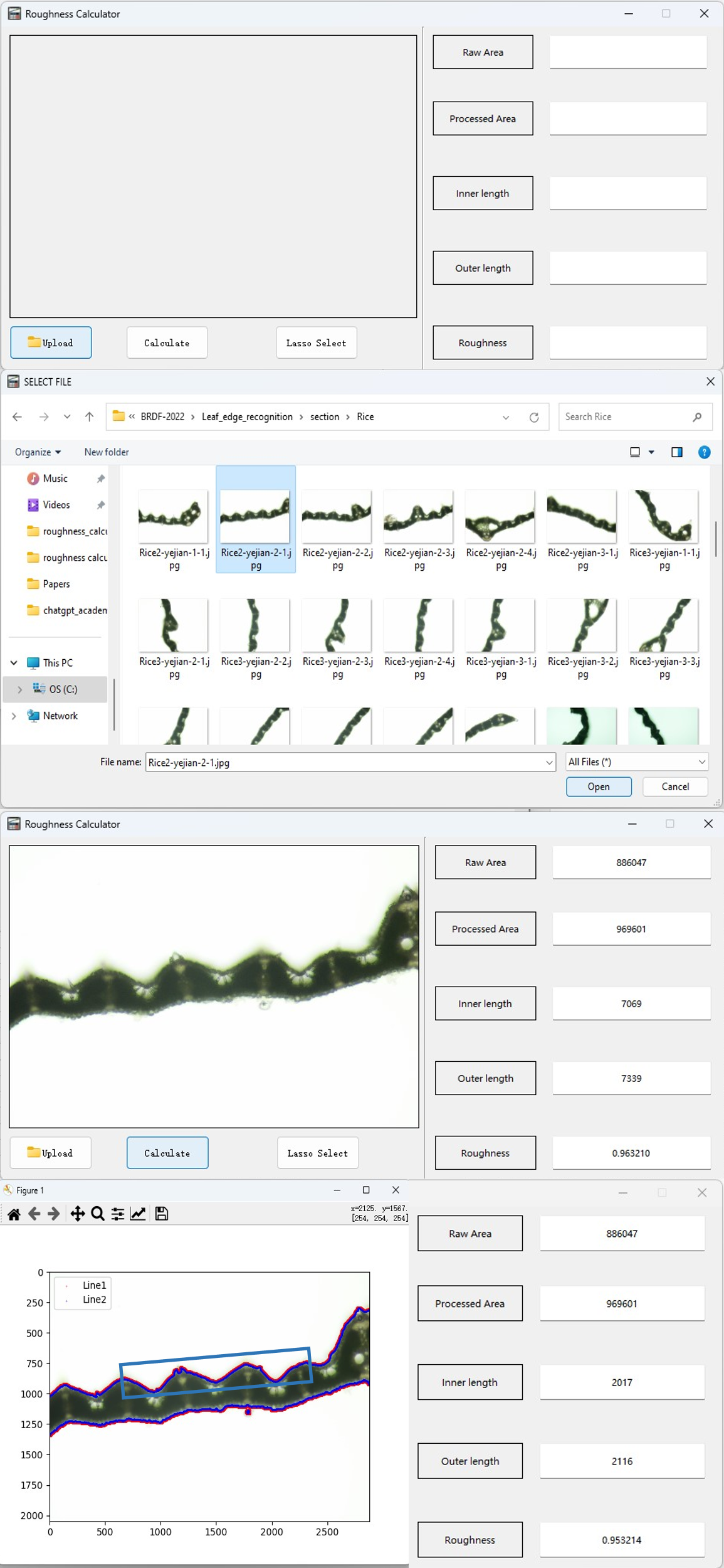


**Figure S4. The interface of the RC.**

We introduce how to use RC based images of leaf sections to calculate the roughness of the leaf surface. The operates as follows:

1. Click "upload", we can select the image from folder in the pop-up window;


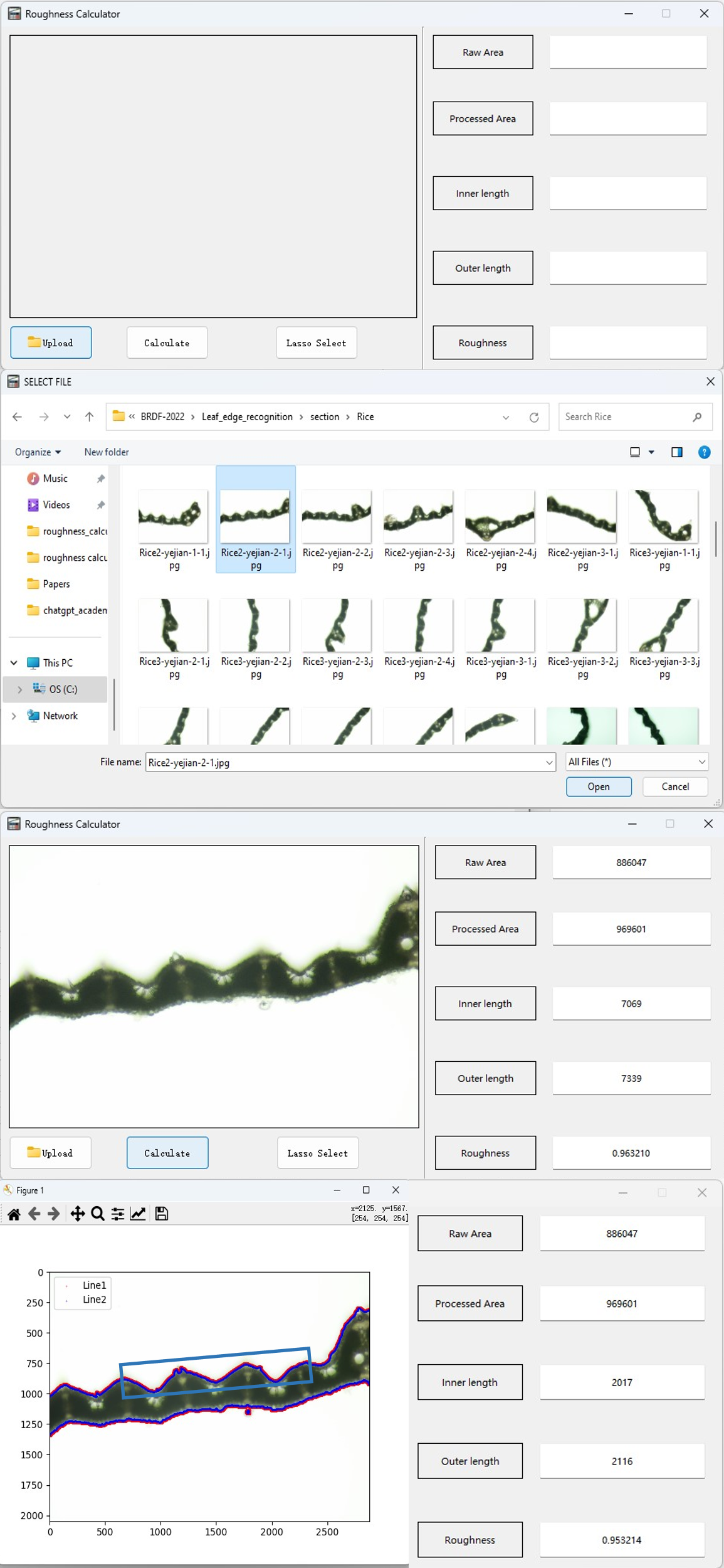


**Figure S5. The pop-up interface of upload window.**

1. Click "calculate", we can obtain the raw area, processed area, inner length, outer length, and roughness of the whole section;


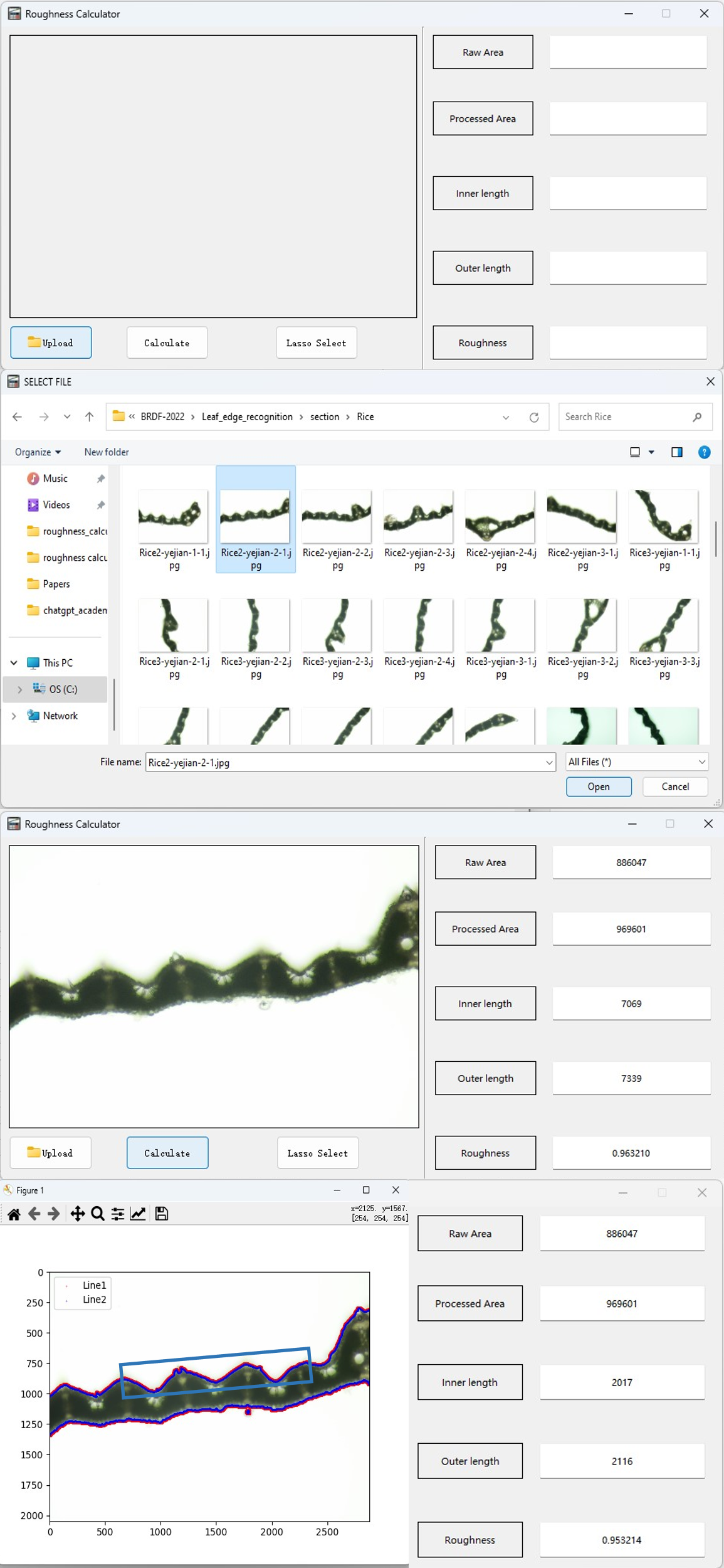


**Figure S6. The interface after calculated parameters of the whole region.**

1. Click "lasso select", we can lasso the region of interest, and the RC will automatically calculate the inner length, outer length, and roughness of the lassoed region.


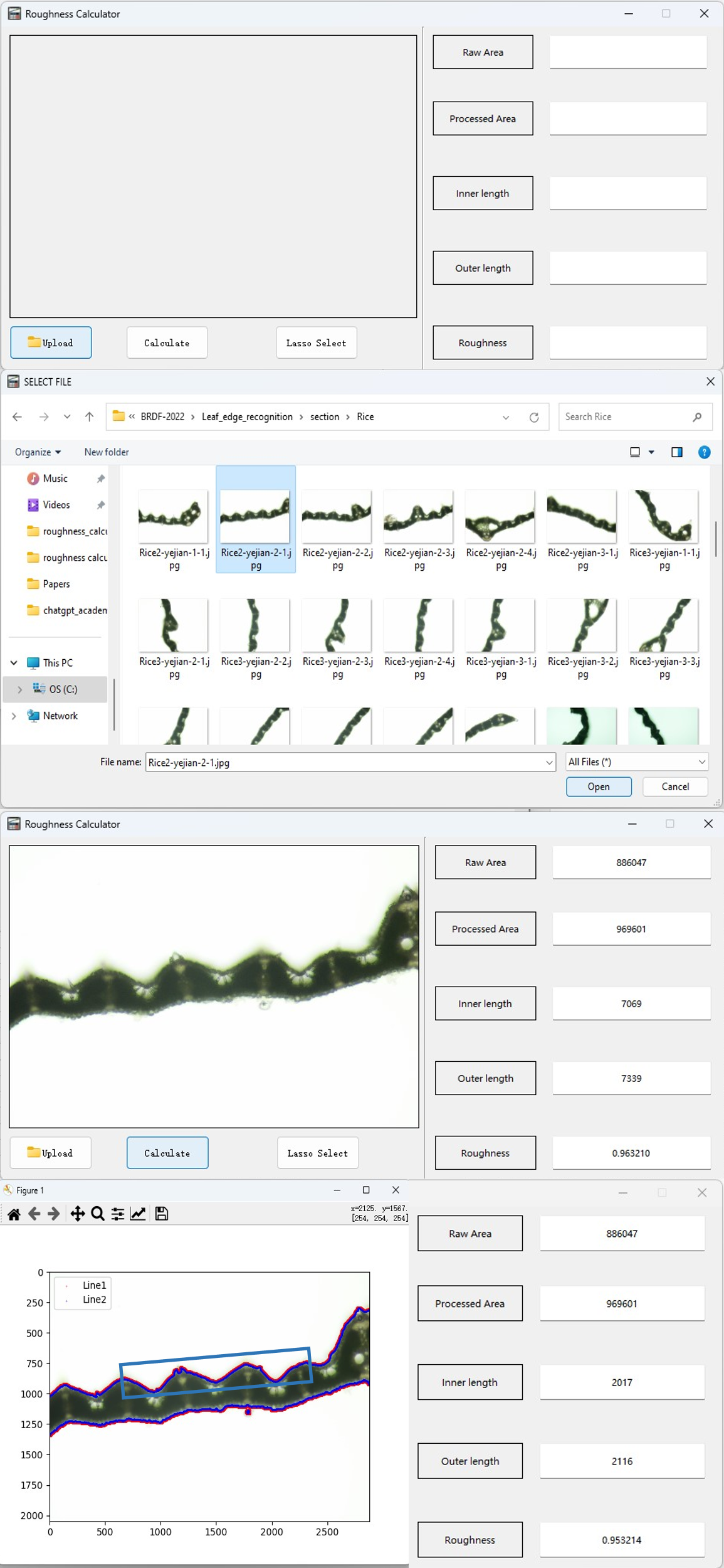


**Figure S7. The interface after calculated parameters of the region of interest.** The red line presents the outer length. The blue line presents the inner length. The baby blue box presents the region of interest.

Notes: A row of buttons above the interface of lasso are reset original view, back to previous view, forward to next view, move view, zoom, configure subplots, edit parameter and save figure.

# **Verification**

The leaf surface of monocots and dicots is quite different; therefore, it was important to evaluate the performance of the software for both types of plants. We analyzed the roughness of monocots (maize and rice) and dicots (poplar and cotton) to evaluate the applicability of the RC. We established a linear regression between the roughness obtained by RC and the leaf roughness parameters fitted by the BRDF model. As shown in Figure S7, the results showed that RC provided a reliable method to quantify the roughness of the leaf surface ($R^{2}>0.82$).


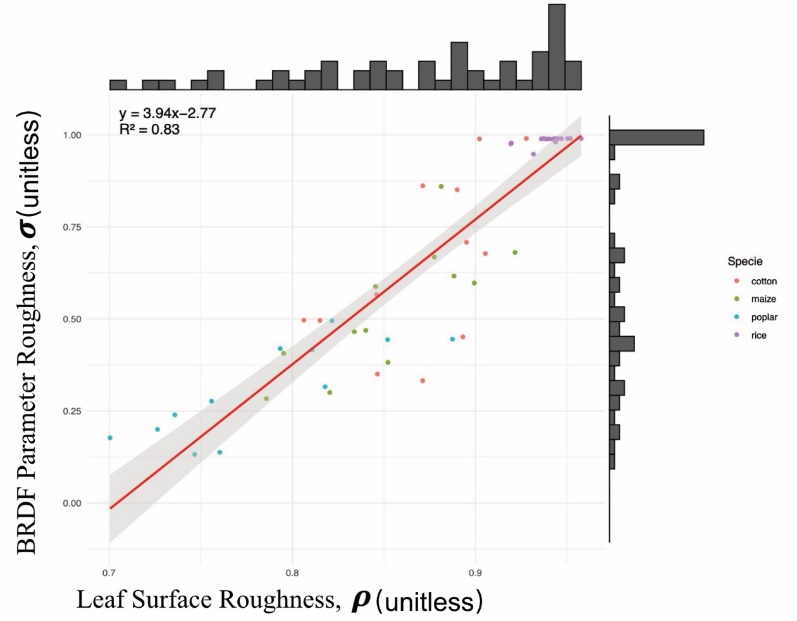


**Figure S8. Correlation between leaf surface roughness (**$\boldsymbol{\rho}$**) and BRDF roughness parameter (**$\boldsymbol{\sigma(\lambda)}$**) for four plant species.** The scatter plot illustrates the linear relationship between leaf surface roughness ($\rho$) and the BRDF roughness parameter ($\sigma(\lambda)$) across four species: cotton, maize, poplar, and rice. Each point represents an individual leaf sample, color-coded by species. The linear regression line, shown in red. The histogram distributions for both $\rho$ and $\sigma(\lambda)$ are displayed along the respective axes.

**Conclusion**

The primary objective of RC is to quantify the roughness parameters in the BRDF model of the leaf. The experimental results show that the measurement results are in good agreement with the actual results. The RC can be used as a tool to quantify the roughness of the blade. It provides the convenient tools for the accurate simulation of light distribution of leaves. In addition to determining the roughness of the leaf surface, the software can also extract other parameters, such as area, length, and circumference, for various objects.


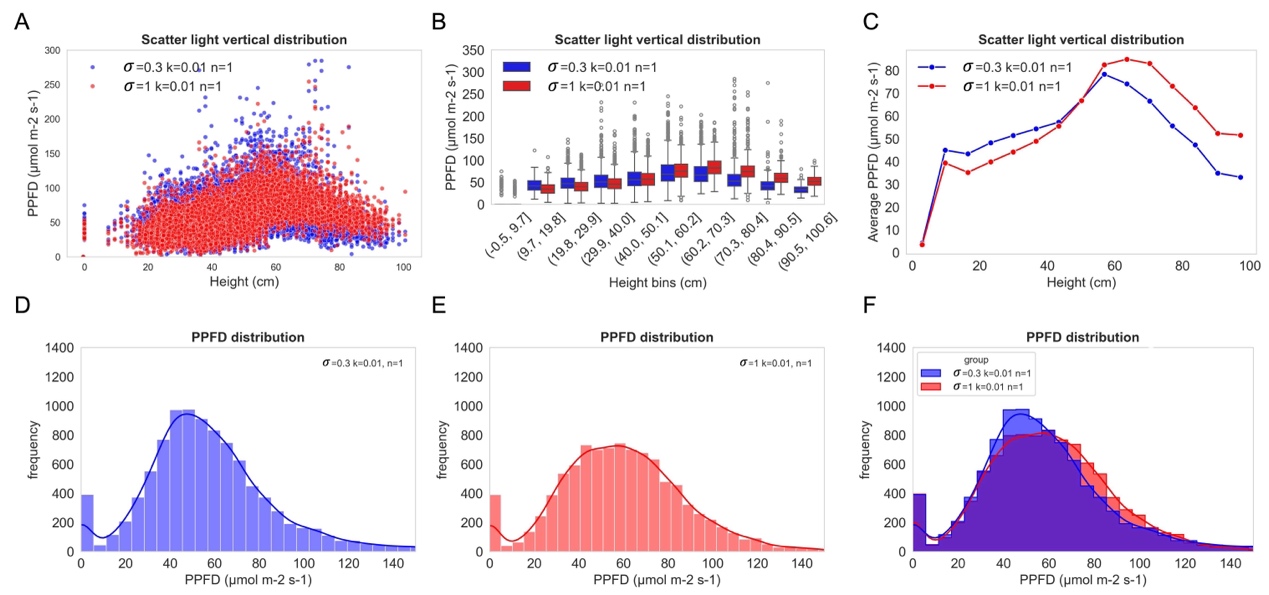


**Figure S9. The impact of leaf roughness (**$\sigma$**) on light distribution within a rice canopy.** (A) Scatter plot of scattered photosynthetic photon flux density (PPFD) versus canopy height. (B) Vertical distribution of scattered PPFD across different canopy layers, presented as box plots. (C) Mean scattered PPFD at each canopy layer. (D, E) Frequency distribution of scattered PPFD under (D) $\sigma$ = 0.3 and (E) $\sigma$ = 1. (F) Comparative histogram of scattered PPFD distributions for both scattering coefficients.

**
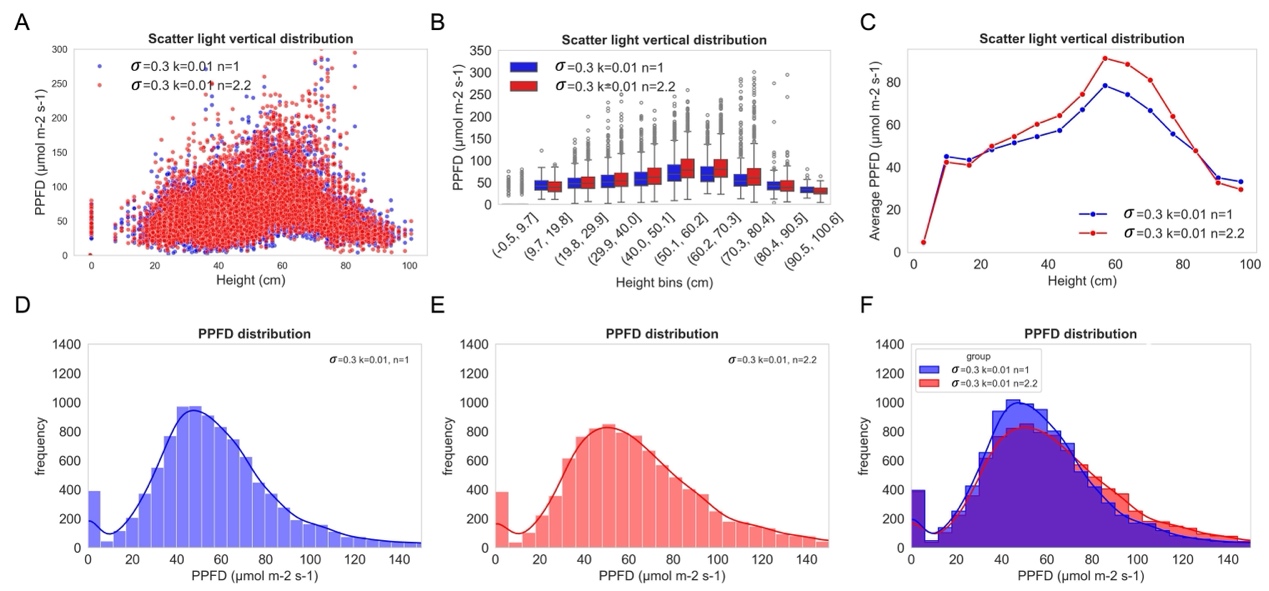
**

**Figure S10. The impact of refractive index (**$\boldsymbol{n}$**) on light distribution within a rice canopy (**$k$ **= 0.01).** (A) Scatter plot of scattered photosynthetic photon flux density (PPFD) versus canopy height. (B) Vertical distribution of scattered PPFD across different canopy layers, presented as box plots. (C) Mean scattered PPFD at each canopy layer. (D, E) Frequency distribution of scattered PPFD under (D) $n$ = 1 and (E) $\sigma$ = 2.2. (F) Comparative histogram of scattered PPFD distributions for both scattering coefficients.


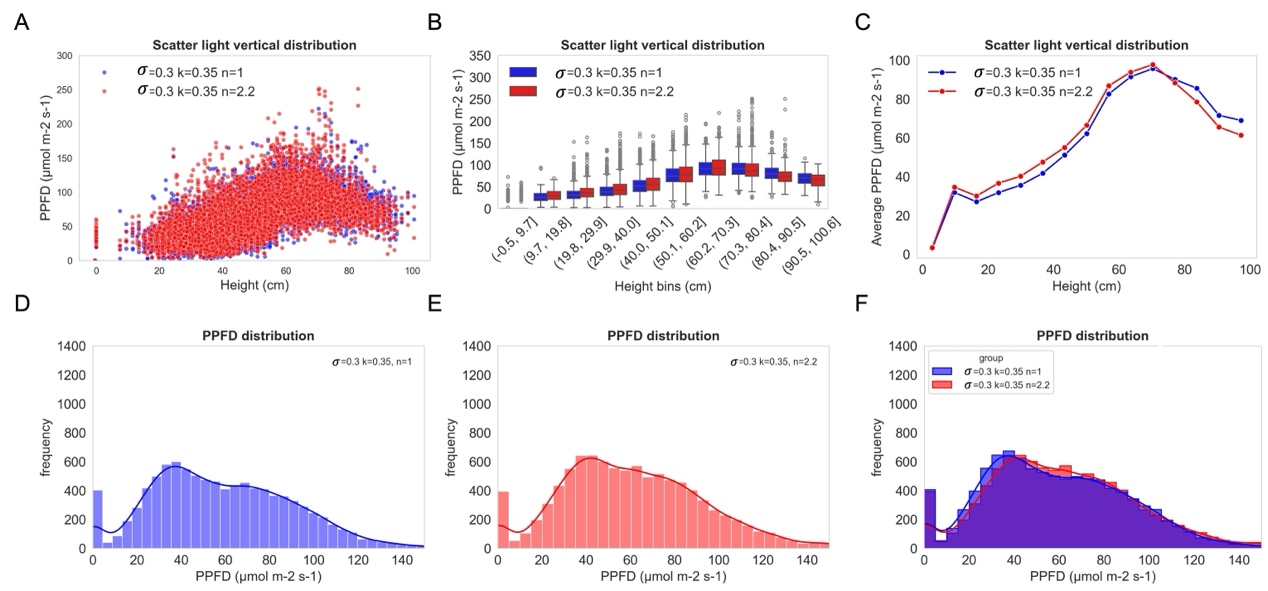


**Figure S11. The impact of refractive index (**$\boldsymbol{n}$**) on light distribution within a rice canopy (**$k$ **= 0.35).** (A) Scatter plot of scattered photosynthetic photon flux density (PPFD) versus canopy height. (B) Vertical distribution of scattered PPFD across different canopy layers, presented as box plots. (C) Mean scattered PPFD at each canopy layer. (D, E) Frequency distribution of scattered PPFD under (D) $n$ = 1 and (E) $n$ = 2.2. (F) Comparative histogram of scattered PPFD distributions for both scattering coefficients.


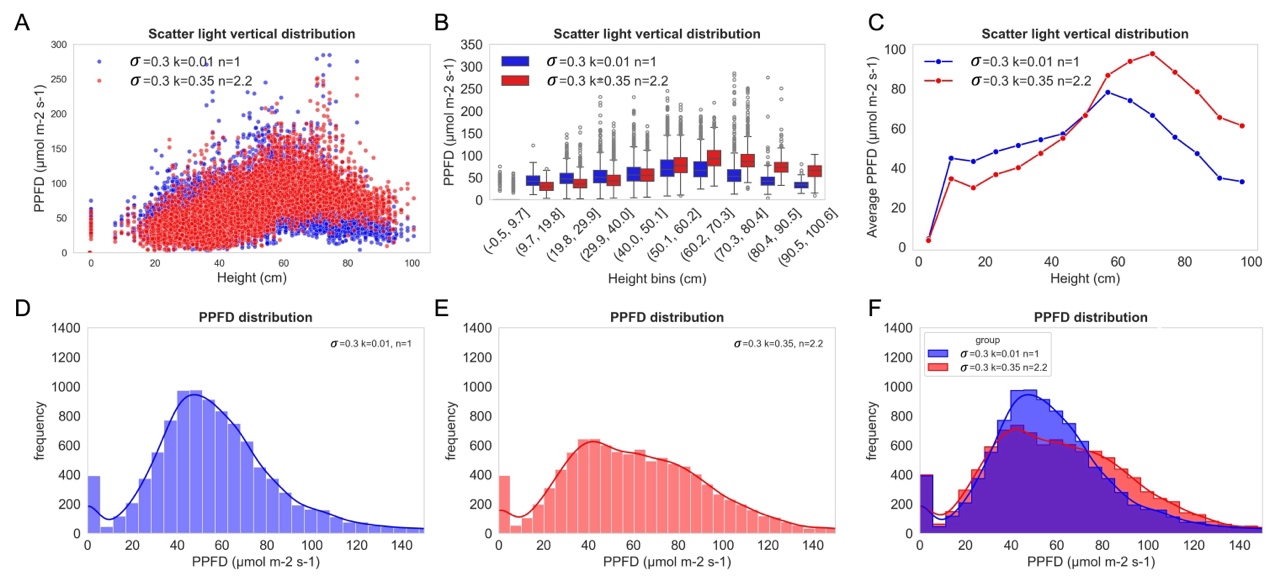


**Figure S12. The impact of diffuse reflection coefficient (**$k$**) and Refractive index (**$\boldsymbol{n}$**) on light distribution within a rice canopy (**$\sigma$ **= 0.3).** (A) Scatter plot of scattered photosynthetic photon flux density (PPFD) versus canopy height. (B) Vertical distribution of scattered PPFD across different canopy layers, presented as box plots. (C) Mean scattered PPFD at each canopy layer. (D, E) Frequency distribution of scattered PPFD under (D) $k$ = 0.01, $n$ = 1 and (E) $k$ = 0.35, $n$ = 2.2. (F) Comparative histogram of scattered PPFD distributions for both scattering coefficients.


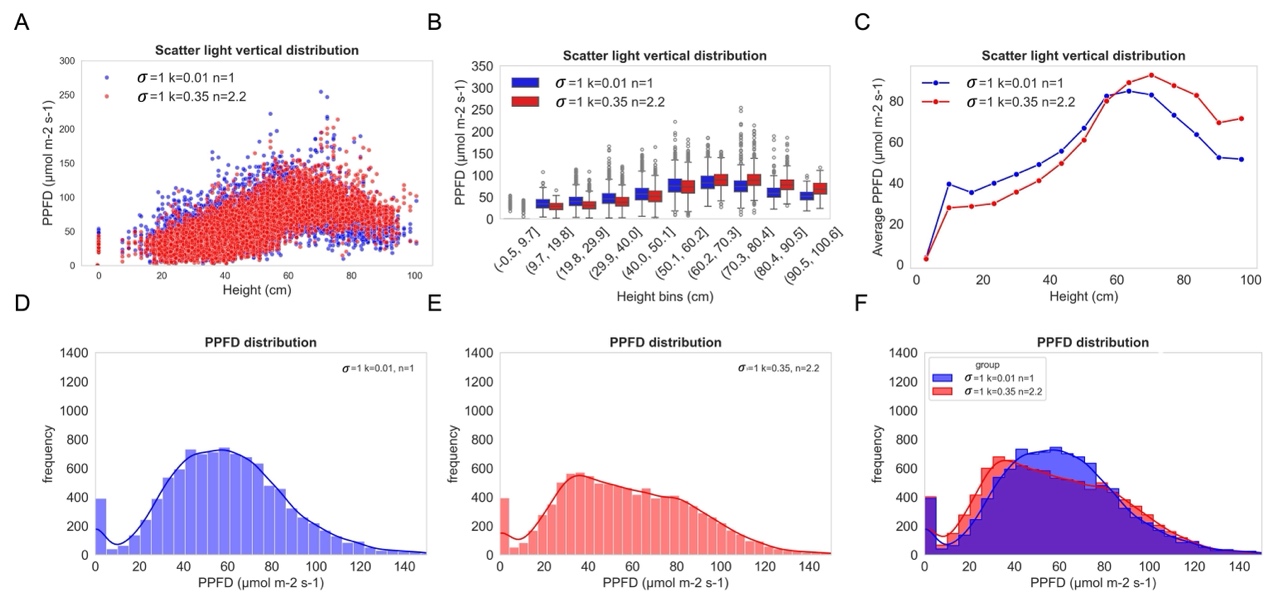


**Figure S13. The impact of diffuse reflection coefficient (**$k$**) and refractive index (**$\boldsymbol{n}$**) on light distribution within a rice canopy (**$\sigma$ **= 1).** (A) Scatter plot of scattered photosynthetic photon flux density (PPFD) versus canopy height. (B) Vertical distribution of scattered PPFD across different canopy layers, presented as box plots. (C) Mean scattered PPFD at each canopy layer. (D, E) Frequency distribution of scattered PPFD under (D) $k$ = 0.01, $n$ = 1 and (E) $k$ = 0.35, $n$ = 2.2. (F) Comparative histogram of scattered PPFD distributions for both scattering coefficients.

**Table S3. Leaf structural and biochemical traits across upper and lower canopy layers for maize, rice, cotton, and poplar.**

| Species | Layers | T  ($\mathrm{mm}$) | SLW  ($g\cdot m^{-2}$) | $Chl.a$  ( $mg\cdot{dm}^{-2}$) | $Chl.b$  ( $mg\cdot\mathrm{dm}^{-2}$) | $Car.$  ( $mg\cdot\mathrm{dm}^{-2}$) |  |
| --- | --- | --- | --- | --- | --- | --- | --- |
| Maize | Upper | 0.17±0.01 | 21.80±2.40 | 2.09±0.81 | 0.52±0.19 | 0.47±0.22 |  |
|  | Lower | 0.19±0.02 | 26.0±0.70 | 4.30±0.46 | 1.14±0.06 | 0.92±0.08 |  |
| Rice | Upper | 0.17±0.02 | 35.90±2.80 | 2.75±0.97 | 0.92±0.35 | 0.69±0.24 |  |
|  | Lower | 0.20±0.01 | 44.70±1.80 | 1.82±0.52 | 0.62±0.17 | 0.55±0.18 |  |
| Cotton | Upper | 0.18±0.01 | 32.00±0.50 | 2.20±0.23 | 0.66±0.06 | 0.47±0.05 |  |
|  | Lower | 0.20±0.02 | 38.90±2.80 | 2.12±0.42 | 0.74±0.13 | 0.66±0.13 |  |
| Poplar | Upper | 0.09±0.01 | 20.30±0.60 | 2.11±0.30 | 0.69±0.06 | 0.55±0.10 |  |
|  | Lower | 0.14±0.03 | 42.00±15.10 | 2.66±0.39 | 0.86±0.16 | 0.70±0.16 |  |
| ANOVA | S | *** | *** | n.s. | n.s. | n.s. |  |
|  | L | *** | *** | n.s. | n.s. | * |  |
|  | S×L | n.s. | n.s. | ** | ** | * |  |

The mean of leaf thickness (T), specific leaf weight (SLW), chlorophyll a ($Chl.a$), chlorophyll b ($Chl.b$), and carotenoids ($Car.$) was ± standard error ($n\geq3$) for different species and leaf levels. Upper represents the upper-layer leaf and Lower represents the lower-layer leaf. T is the thickness of the leaf in $\mathrm{mm}$; SLW is the specific leaf weight in $g\cdot m^{-2}$; $Chl.a$, $Chl.b$, and $Car.$ are the contents of chlorophyll a, chlorophyll b and carotenoids in $mg\cdot\mathrm{dm}^{-2}$. In the ANOVA results, S represents the main effect of Species, L represents the main effect of Layer, and S × L represents the interaction between species and layers. The significance level is: *** for p < 0.001, ** for p < 0.01, * for p < 0.05 and n.s. for p > 0.05 respectively.
